# Hydrogel muscles powering reconfigurable micro-metastructures with wide-spectrum programmability

**DOI:** 10.1038/s41563-023-01649-3

**Published:** 2023-08-21

**Authors:** Mingchao Zhang, Aniket Pal, Zhiqiang Zheng, Gaurav Gardi, Erdost Yildiz, Metin Sitti

**Affiliations:** 1 Physical Intelligence Department, Max Planck Institute for Intelligent Systems, Stuttgart, Germany; 2 Institute for Biomedical Engineering, ETH Zürich, Zürich, Switzerland; 3 School of Medicine and College of Engineering, Koç University, Istanbul, Turkey

**Keywords:** Gels and hydrogels, Information storage

## Abstract

Stimuli-responsive geometric transformations endow metamaterials with dynamic properties and functionalities. However, using existing transformation mechanisms to program a single geometry to transform into diverse final configurations remains challenging, imposing crucial design restrictions on achieving versatile functionalities. Here, we present a programmable strategy for wide-spectrum reconfigurable micro-metastructures using linearly responsive transparent hydrogels as artificial muscles. Actuated by the hydrogel, the transformation of micro-metastructures arises from the collaborative buckling of their building blocks. Rationally designing the three-dimensional printing parameters and geometry features of the metastructures enables their locally isotropic or anisotropic deformation, allowing controllable wide-spectrum pattern transformation with programmable chirality and optical anisotropy. This reconfiguration mechanism can be applied to various materials with a wide range of mechanical properties. Our strategy enables a thermally reconfigurable printed metalattice with pixel-by-pixel mapping of different printing powers and angles for displaying or hiding complex information, providing opportunities for encryption, miniature robotics, photonics and phononics applications.

## Abstract (web summary)

It is difficult to program a single stimuli-responsive geometry to transform into diverse final configurations in a systematic manner. Here, linearly responsive transparent hydrogels are developed to create micro-metastructures with wide-spectrum thermal reconfigurability.

## Main

Metamaterials are a category of artificial materials composed of periodically arranged basic units, which enable exotic properties beyond their natural and bulk counterparts^1–3^, such as tuneable mechanical properties^4^, negative refractive index^5^ and negative Poisson’s ratio^6^. Compared to their conventional passive and static behaviours, geometric transformations of metamaterials responding towards external stimuli^7–9^, such as light^10^, heat^11^, hydration^12^ and magnetic, electrical and electrochemical fields^13–15^, attracts wide interdisciplinary interest for their stimuli-responsive, adaptive and programmable properties and functions^16^. Translating the programmable actuated metastructures into the microscale or nanoscale will provide great opportunities for developing high-density functional subunits^12,15^ that advance the development of laboratory-on-a-chip technologies^17^, microrobots^18^, physically intelligent machines^19^, low-energy mechanical computation^20^ and tuneable photonic and phononic devices^21,22^. Their miniaturization, however, is challenging due to the limitation of actuation mechanisms at the micro- and/or nanoscale that usually work exclusively for a specific material^23^. Moreover, metamaterials with a certain initial geometry usually transform into a specific final configuration^11,12^, and different final configurations only can be generated by designing other initial geometries^15^. It is challenging to realize programmable and diverse configurations that are transformed all from the same initial geometry. For example, certain prescribed cuts in the kirigami metastructures exclusively generate a specific final pattern, while other configurations only can be achieved by redesigning different types of cut (that is the joint to cut ratio)^11,24^. The lack of wide-spectrum programmability of metastructures tremendously restricts their design diversity, functions and real-world applications.

Among different transformation mechanisms, artificial muscles stand out as a potential actuation method for reconfigurable metamaterials because of their strong actuation force, fast temporal responsiveness and high power density^11^. Several properties of hydrogels^25–29^, such as their benign aqueous environment, easily tailored chemical composition and properties^30,31^, and softness compatible with biological tissues, make them attractive as artificial muscles for soft robotics, implantable devices and biomedical applications^32–35^. To achieve reconfigurable metastructures, hydrogel muscles possessing properties such as large, transparent and uniform deformation is crucial^31^. Linear response ensures uniform actuation of the metastructures without causing structural damage or failure, whereas the high maintenance in the transparency of the hydrogel muscles allows the optical observation of the metastructures during the transformation process.

**Fig. 1:**
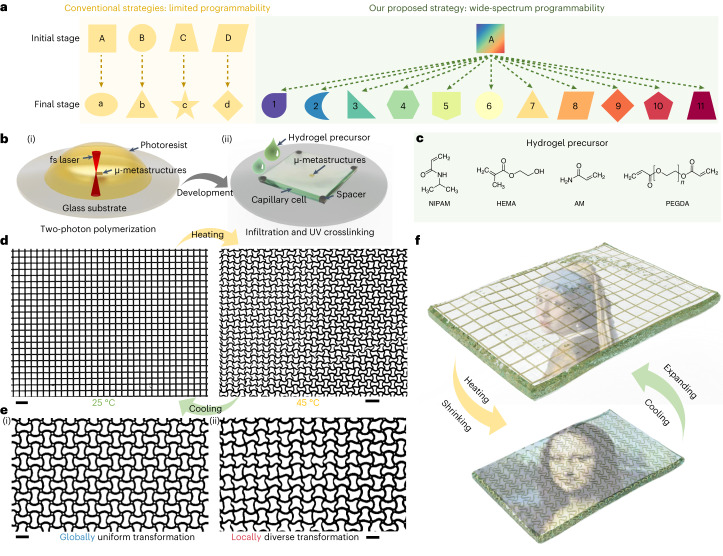
Concept of the proposed wide-spectrum programmability and the strategy of the reconfigurable micro-metamaterials actuated by a linearly responsive hydrogel muscle (LIHAM). **a**, Schematic illustration showing the comparison between the limited programmability through conventional approaches and the wide-spectrum programmability through our proposed strategy with programmable shape and optical polarization (represented by colours), for reconfiguring the building blocks of metastructures. **b**, Schematic illustration showing the fabrication steps of the reconfigurable micrometre-scale metastructures: (i) two-photon polymerization-based micropatterning of the photoresist, and (ii) infiltration and UV cross-linking of the linearly responsive hydrogel precursor. **c**, Main precursors of the LIHAM. **d**, Optical microscope images showing a typical as-printed metalattice composed with locally different laser powers and its transformed pattern before and after heating. Scale bars, 20 μm. **e**, Optical microscope images showing the globally uniform (i) and locally diverse (ii) transformation of the metalattice. Scale bars, 10 μm. **f**, Schematics of the reversible thermal actuation of the micro-metastructures displaying dynamic encrypted complex information.

Here we propose an actuation mechanism based on a linearly responsive, transparent hydrogel as artificial muscles for reconfigurable micro-metastructures and achieve a wide pattern-transformation spectrum. The reconfigurability of such micro-metastructures comes from the different reversible geometric transformation states that we can control by changing the actuation temperature. Our strategy offers substantial design diversity on reconfigurable two-dimensional (2D) and three-dimensional (3D) miniature metastructures with tuneable chirality and optical anisotropy, and it can be applied to a variety of other 3D-printable metastructures and materials. In particular, conventional actuation strategies limit the building blocks of metamaterials to transform from a specific initial geometry to a single, predetermined final configuration, and achieving different final configurations requires designing different initial geometries (Fig. 1a). By contrast, our approach achieves a wide pattern-transformation spectrum by continuously changing the local printing parameters of the pixelated building blocks, enabling the same initial geometry of the building blocks to be reconfigured into a wide range of geometries. Additionally, our approach allows for incorporating additional functionalities, such as reconfigurable optical anisotropy, into the pixelated building blocks, greatly enriching the design diversity and increasing the complexity of functionalities that can be achieved in the field of reconfigurable metastructures.

## Concept of the reconfigurable micro-metastructures

**Extended Data Fig. 1:**
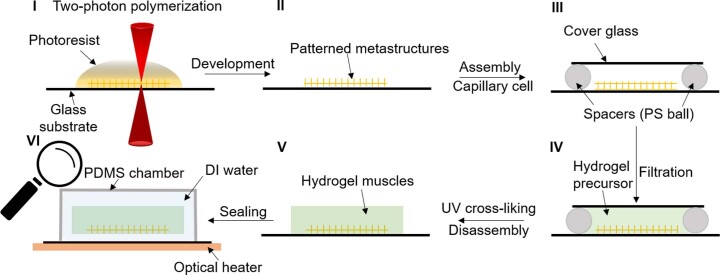
Schematics showing the fabrication steps of the reconfigurable micro-scale metastructures. (**I**) two-photon polymerization (2PP) based micropatterning of the photoresist on a glass substrate; (**II**) developing in solvents (DI water, acetone, and 2-propanol for gelatin, DEGRAD INX N100, and IP-S photoresist, respectively); (**III**) assembly of a capillary cell above the metastructures using polystyrene balls with desired thickness coated with UV glue as the spacers, followed by 1 min of UV exposure; (**IV**) filtration of hydrogel precursor solution into the capillary cell, followed by 15 min of UV exposure; (**V**) disassembly of the cover glass from the capillary cell and sealing in a PDMS chamber; (**VI**) releasing of the hydrogel muscle from glass substrate by heating before any measurement.

The strategy for the reversible reconfigurability of micro-architected metamaterials relies on their actuation by a linearly responsive hydrogel artificial muscle (LIHAM). The soft hydrogels can contract, like muscles, to compress embedded skeletons made of the two-photon polymerized (2PP) photoresists. Transformable 2D or 3D metastructures are fabricated on a glass substrate via the 2PP-based 3D microprinting technique with a resolution down to 100 nm (Fig. 1b). After development, a capillary glass cell is assembled above the patterned metastructure spaced by polystyrene balls on each corner for achieving a desired thickness. Then, the hydrogel precursor (Fig. 1c) is infiltrated into the capillary cell, and the micro-metastructure is embedded into the cross-linked hydrogel by ultraviolet (UV) treatment (details in Methods and Extended Data Fig. 1). The shrinkage and expansion of the hydrogel, induced by heating and cooling, respectively, enable the reversible transformation of the printed metastructures. The shrinkage of the hydrogel forces the printed beams, the basic unit of these metastructures, to buckle into an in-plane sinusoidal profile. The cooperative buckling of the intersecting beams allows the transformation to a new architecture (Fig. 1d). In particular, this strategy can enable the global transformations of metastructures into another uniform configuration (Fig. 1e(i) and Supplementary Fig. 1), as well as allow to locally program each pixelized building block for reversible and site-specific transformations into versatile configurations (Fig. 1e(ii)). With such a wide-spectrum programmability, it is possible to encrypt highly complex information, such as the paintings of the *Mona Lisa* and the *Girl with a Pearl Earring*, within the metastructures. The thermal actuation of LIHAM can enable the reconfiguration of the metastructures to display or hide this encrypted information, as depicted in Fig. 1f.

## Synthesis of the LIHAM

**Fig. 2:**
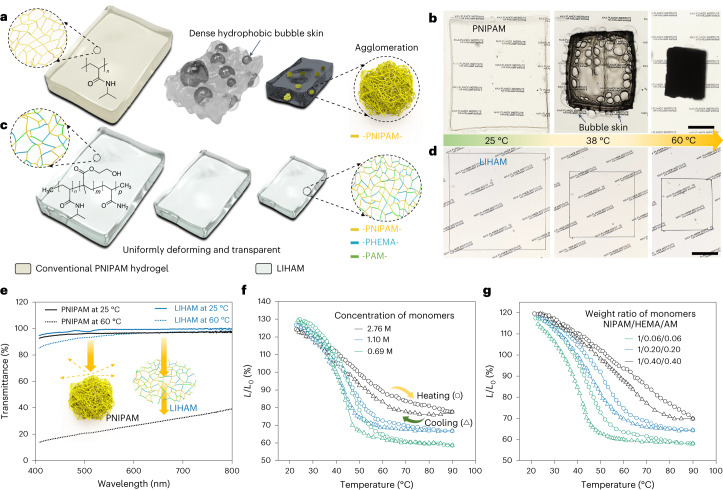
LIHAM and its comparison with a conventional thermally responsive hydrogel (PNIPAM). **a**, Schematic illustration of the nonlinear deformation of conventional pure PNIPAM hydrogel. **b**, Optical images showing the shrinking process of the PNIPAM hydrogel. **c**, Schematic illustration of uniform deformation while maintaining the transparency of the LIHAM. **d**, Optical images showing the shrinking process of the LIHAM. **e**, Transmittance of the PNIPAM and LIHAM before and after heating, inset shows the schematics illustrating the mechanism of their transparency. **f**,**g**, Effect of concentration of monomers (**f**) and their weight ratio (**g**) on the deformation of the LIHAM. Scale bars, 500 μm. Source data

Large, transparent and uniformly deforming hydrogels are crucial for the construction of reconfigurable metastructures. Poly(*N*-isopropylacrylamide) (PNIPAM) has been widely used for constructing various stimuli-responsive systems^36^. The deformation of the PNIPAM hydrogel is driven by the desorption and absorption of water, induced by the temperature-mediated hydrophilic–hydrophobic transition of the polymer chains (Fig. 2a). The transition, however, usually results in a catastrophic collapse of the network chains, forming dense hydrophobic agglomerates^37^, which act as hydrophobic barriers and prevent the smooth diffusion of water, slowing down their deformation process. As a consequence, dramatic morphological changes (for example, bubble skin) due to their nonlinear deformation can be seen (Fig. 2b). Moreover, the agglomeration causes scattering of the incident light, reducing the transparency of the hydrogel (Supplementary Video 1).

To solve these issues, while we adopted many strategies to tailor the PNIPAM hydrogel including the interpenetrating polymer network strategy (details in Methods, Supplementary Fig. 2 and Supplementary Video 2), we finally used a terpolymerization strategy. Two hydrophilic monomers, namely 2-hydroxyethyl methacrylate (HEMA) and acrylamide (AM), were copolymerized with *N*-isopropylacrylamide (NIPAM) monomers, while polyethylene glycol diacrylate (PEGDA) served as a flexible cross-linker (Fig. 1c). In contrast to pure PNIPAM hydrogel, the synthesized poly(NIPAM-*co*-HEMA-*co*-AM) hydrogel shows very uniform deformation while maintaining high transparency (Fig. 2c,d and Supplementary Video 3). The LIHAM constitutes of reinforced hydrophilic polymer chains, which strongly support and strengthen the network system, preventing the collapse of PNIPAM polymer networks during the temperature-mediated hydrophilic and hydrophobic transition. As a result, these reinforced polymer chains uniformly shrink without generating light-scattering agglomerates, thus maintaining the transparency of the LIHAM. Figure 2e compares the transmission (*λ* = 580 nm) of the polymers when heated to 60 °C, showing a negligible loss for the LIHAM (3.4%) compared to the substantial loss for the PNIPAM (71.2%) (Supplementary Fig. 3). Besides, the deformation abilities of the LIHAM can be tuned by adjusting the concentration of the monomers (Fig. 2f) and their weight ratio (Fig. 2g) (see the details in the Methods).

## Transformation mechanism

**Fig. 3:**
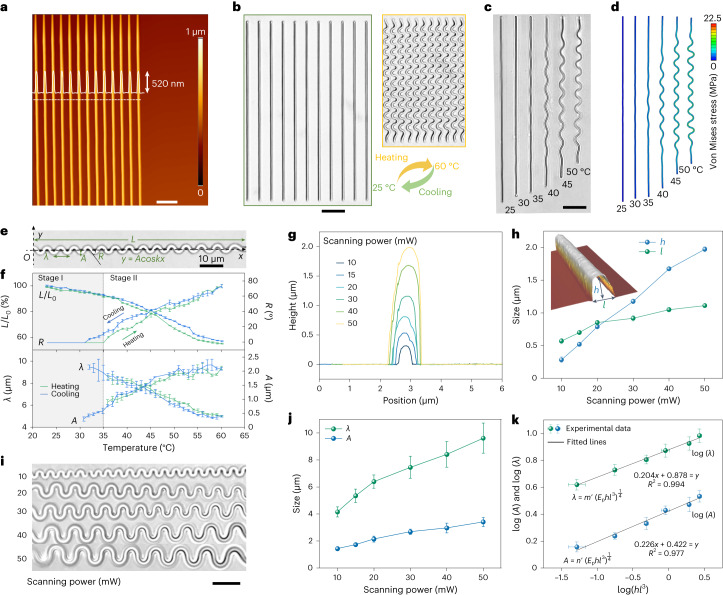
Mechanism of the metastructure reconfiguration. **a**, AFM image of the 3D-printed beam arrays. Scale bar, 5 μm. **b**, Optical microscope images of the printed beam arrays before and after heating. Scale bar, 20 μm. **c**,**d**, Snapshots showing the experimental (**c**) and FEA (**d**) results of the transformation of a printed beam at different temperatures. Scale bar, 20 μm. **e**, Optical microscope image of the bulked beam denoted by *y* = *A*cos*kx*. **f**, Evolution of the geometric parameters of the printed beams at different temperatures. Data points are shown as mean ± s.d. (*n* = 6). **g**, Cross-section profiles of the beams printed with different printing powers. **h**, Characteristic size of the cross-section of different beams printed through at different scanning powers. The inset is a 3D morphology obtained by AFM. Data points are shown as mean ± s.d. (*n* = 6). **i**, Optical images showing morphologies of the buckled beams at 60 °C printed with different scanning powers. Scale bar, 10 μm. **j**, Wavelengths and amplitudes of buckled beams under different scanning powers. Data points are shown as mean ± s.d. (*n* = 18). **k**, Relationship of the cross-sectional parameters of the printed beams and their geometrical parameters of the buckled beams. Data points are shown as mean ± s.d. (*n* = 18). Source data

To understand the transformation behaviours of the LIHAM-actuated micro-metastructures, we recorded and analysed the evolution of the constituent building block of our system, that is, a straight beam with a rectangular cross-section. Figure 3a shows the atomic force microscopy (AFM) image of an array of beams with submicrometre resolution, where both the topology and cross-section of the beams show reproducibility and no discernible variation between them. When the 3D-printed beams are embedded into the LIHAM hydrogel matrix and exposed to heat, the hydrogel experiences isotropic volume contraction, while the printed structures do not. This differential contraction causes the beams to buckle and adopt a uniform, sinusoidal profile (Fig. 3b,c). On the removal of heat, as the hydrogel expands and reverts to its original size, the printed beams also recover their initial linear profile. We performed nonlinear finite element analysis (FEA) to further investigate the buckling behaviour of the printed beams (see Methods for more details). Figure 3d shows the evolution of the buckling-based deformation of a printed beam (50 mW power) due to heating, which matches well with the experimental observations.

We investigated the shape evolution of a printed beam at different temperatures to understand how its geometrical parameters change (Fig. 3e,f, Supplementary Fig. 4 and Supplementary Video 4). A cosine function (*y* = *A*cos*kx*, where *k* = 2π/*λ*) was used to quantitively describe the transformation process, and the corresponding wavelength (*λ*), amplitude (*A*), rotation angle of nodes (*R*) and axial length (*L*) were measured (Fig. 3e). As shown in Fig. 3f, the beam experiences two deformation stages during the heating process: uniform compression (stage I) and buckling (stage II). No appreciable wave architecture can be seen during stage I (no values of *λ*, *A* and *R*), while the axial length (*L*) shrank uniformly. After reaching a threshold temperature, the beam suddenly buckled into a detectable wave architecture; values of *A* and *R* increased, while *λ* and *L* continued to decrease as the temperature rose during stage II. After cooling, these parameters recovered, albeit with a small hysteresis, which resulted from the relaxation of polymer networks. To understand the in-plane buckling-induced transformation, an analytical model based on surface elastic theory was used^38^ (see the details in Methods). By the minimization of the total potential energy for the in-plane buckling, we obtained the analytical solutions of the characteristic geometries. As the results indicate, these geometries are determined by the ratio of the elastic moduli of the printed beams (*E*_p_) and LIHAM matrix (*E*_0_), as well as the bending stiffness of the beams.

As demonstrated by the analytical model, the buckled geometries of the reconfigurable metastructures strongly depend on the cross-sectional geometries of the printed beams that are determined by the 3D-printing parameters (scanning laser power and scanning speed) during the 2PP fabrication process (Fig. 3g). As shown in Fig. 3h, the width (*l*) and height (*h*) of the cross-section of the printed beams increase with the scanning power. As a result, with an increase in scanning power, both the wavelength (λ) and amplitude (*A*) increased (Fig. 3i,j). To validate the model and determine how these printing parameters affect the final configuration, we fitted these experimental data. As shown in Fig. 3k, good linear relationships (*R*^2^ = 0.994 for *λ*, and *R*^2^ = 0.977 for *A*) can be obtained, demonstrating the validity of the model. The scanning speed of the 2PP process also influences the cross-section profiles of the printed beams (Supplementary Fig. 5a,b), but its influence on their geometry and final buckled configuration is not as remarkable as that of the scanning power (Supplementary Fig. 5c). In addition, this reconfigurable strategy can be applied to other metastructures with different material compositions and geometries. To prove it, both very soft (GelMa^39^) and rigid (IP-S) photoresists could be used to construct the reconfigurable metamaterials (Supplementary Fig. 6). Here, the shape-morphing behaviour of any metastructures would be a function of both material elastic modulus and structure geometry (see the Analysis of the LIHAM-actuated shape-morphing behaviours section in the Methods for further details).

## Design principle for 2D and 3D reconfigurable metastructures

**Fig. 4:**
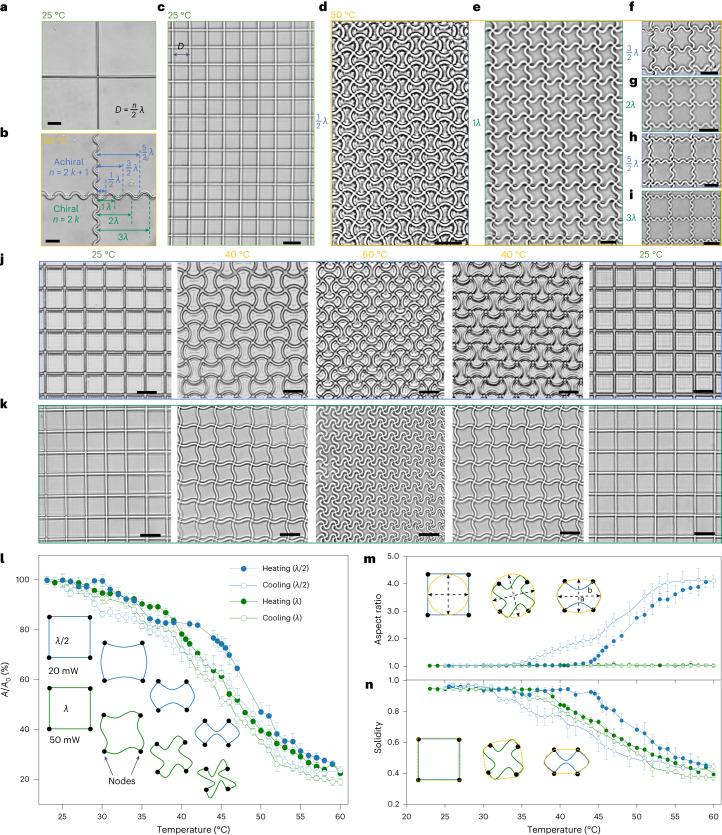
Design and transformation of the reconfigurable metastructures. **a**, Optical image of a printed cross at 25 °C. **b**, Optical image showing the transformation of the printed cross at 50 °C. **c**, Optical image of a printed square lattice. **d–i**, Optical image showing the transformation of different chiral and achiral metastructures. The achiral metastructures belong to the metalattices with hatching distances (*D*) being an odd multiple (*n* = 2*k* + *1*) of the half-wavelength (*λ*/2) of the transformed structures (that is, *D* = (2*k* + 1)*λ*/2), such as *n* = 1 (**d**), *n* = 3 (**f**) *n* = 5 (**h**) and the chiral metastructures belong to the metalattices with *D* being an even multiple (*n* = 2*k*) of the half-wavelength (λ/2) of the transformed structures (that is, *D* = 2*kλ*/2), such as *n* = 2 (**e**), *n* = 4 (**g**) and *n* = 6 (**i**). **j**–**k**, Snapshots showing the achiral (**j**) and chiral (**k**) transformation of two metalattices with the same *D* of 10 µm. **l**–**n**, Area change (**l**), aspect ratio (**m**) and solidity (**n**) of the unit cells showing the achiral and chiral transformation of the two metalattices as a function of temperature. Data points are shown in **l**–**n** as mean ± s.d. (*n* = 50). Scale bars, 10 μm. Source data

On the basis of the above transformation behaviours of the printed beams, we realized the construction of reconfigurable 2D micro-metastructures. To illustrate the design principle, we printed two perpendicularly intersecting beams (Fig. 4a), which, after heating, buckled into two mutually intersecting sinusoidal profiles (Fig. 4b). The successful construction of reconfigurable metastructures should follow the rule: the distance between two adjacent beams, that is, the hatching distance (*D*) should be an integer multiple (*n*) of half-wavelength (*λ*/2) of the wave structure ($D=\frac{n}{2}\lambda$). In other words, the distance between neighbouring nodes should contain multiple half-wavelength structures. In this way, the printed metalattices (Fig. 4c) could transform into a new morphology without generating defects (Fig. 4d–i, an FEA 2D result referring to Supplementary Fig. 7). Otherwise, non-uniform stresses would result in defective patterns and cause structural failure (Supplementary Fig. 8).

It should be noted that the intersected beams rotate by the same angle and direction around each node to minimize elastic energy, which allowed the chirality of the buckled patterns to be manipulated. For example, in a square lattice, when the prescribed *n* was an odd multiple (*n* = 2*k* + 1, where *k* is an integer) of half-wavelength (*λ*/2), the buckled pattern was achiral (Fig. 4d,f,h), while *n* was an even multiple (*n* = 2*k*), it was chiral (Fig. 4e,g,i). After chiral transformations, each of the adjacent unit cells was identical but geometrically asymmetrical. By contrast, for achiral transformations, the orientations of adjacent unit cells were mutually vertical and each of them was symmetrical.

With different scanning powers, metalattices with the same initial lattice (*D* = 10 µm) could transform into different morphologies. As shown in Fig. 4j and Supplementary Video 5, a lattice fabricated with a scanning power of 50 mW was reversibly transformed into a new morphology with a half-wavelength (*λ*/2) between two adjacent nodes. By contrast, a lattice with the same lattice constant, fabricated with a scanning power of 20 mW morphed into a different morphology with a one wavelength (λ) between two neighbouring nodes (Fig. 4k and Supplementary Video 6). During actuation, the areas of the unit cells of both lattices changed by a similar degree (Fig. 4l), while their geometric transformation varied notably such as aspect ratio (Fig. 4m) and solidity (Fig. 4n) (see details in the Methods). The cyclic actuation tests for both modes demonstrate a strong and robust interface between the metastructures and LIHAM, as indicated by their good actuation stability during the cyclic heating and cooling tests (Supplementary Fig. 9). Besides, the minimum addressable size of a unit cell of the metastructures could be roughly 3.3 μm on the basis of the current 2PP process (Supplementary Fig. 10), reaching a high cell density of about ten million per square centimetre. Additionally, the current 2PP method can readily be expanded to create macroscopic reconfigurable metastructures with microscopic subunits (for example, 1.2 × 1.2 cm^2^, Supplementary Figs. 11a–c), and our actuation strategy is also compatible with large-scale metastructures manufactured using UV lithography.

**Extended Data Fig. 2:**
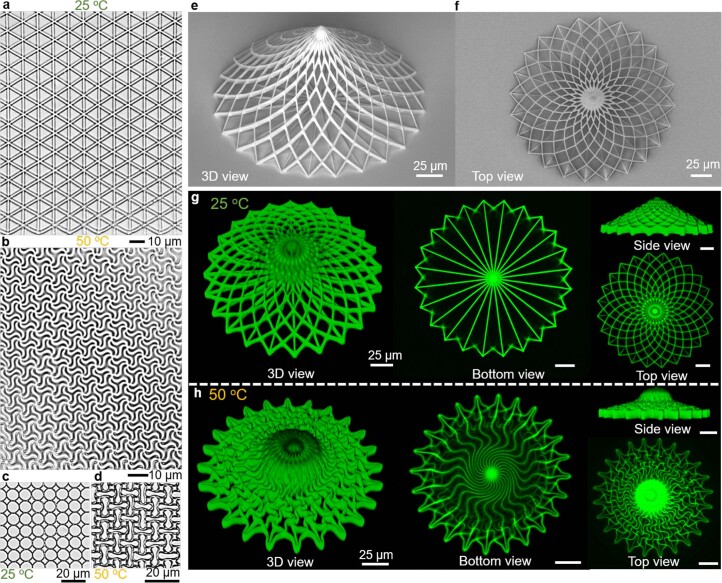
Experimental demonstration of versatile 2D and 3D reconfigurable metastructures. **a**, Optical image of a printed triangular metalattice in the LIHAM matrix at 25 °C. **b**, Optical image showing the geometric transformation of the triangular meta-lattice at 50 °C. **c**, Optical images showing the geometric transformation of a printed circular metastructure in the LIHAM matrix at 25 °C. **d**, Optical image showing the geometric transformation of the circular metastructures at 50 °C. **e**, Scanning electron microscopy (SEM) image showing the 3D view of a tent-like architecture. **f**, SEM image showing the top view of the tent-like 3D architectures. **g-h**, Confocal fluorescent microscopes showing the 3D view, bottom view, side view, and top view of the tent-like 3D architectures (printed with IP-S) at 25 °C (**g**) and 50 °C (**h**). Scale bars, 25 µm.

On the basis of the above design principles, we could realize a large variety of reconfigurable 2D and 3D complex metastructures. For example, a triangular metalattice (Extended Data Fig. 2a) could transform into a very different morphology on stimulation (Extended Data Fig. 2b, Supplementary Fig. 12 and Supplementary Video 7), and circle-array metastructures could turn into a new pattern composed of mutually vertical ellipses (Extended Data Fig. 2c,d), while varying the scanning power along different directions of a lattice could generate locally different and inhomogeneous geometric transformations (Supplementary Fig. 13). Besides, the transformation strategy could be extended to 3D architectures, so we printed a 3D tent-like architecture composed of high-aspect-ratio beams (Extended Data Fig. 2e,f). To observe its reconfigurable transformation, we used a confocal fluorescent microscope to record their 3D architectures at low and high temperatures, respectively (Extended Data Fig. 2g,h). The tent-like architecture including a cone and radial bottom (Extended Data Fig. 2g) transformed into a new architecture with constituent buckled beams at 50 °C (Extended Data Fig. 2h and Supplementary Video 8), enabling a clockwise rotation to the bottom and intricate morphology to the cone. Therefore, we believe the extension of the deformation mechanism to 3D architectures will widen the design flexibility and motivate more promising applications.

## Wide-spectrum programmability for information encryption

**Fig. 5:**
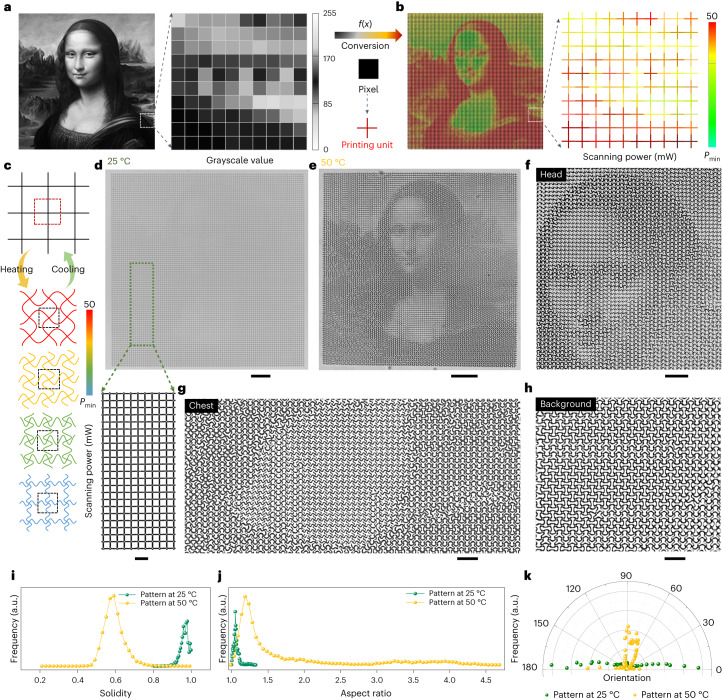
Proof-of-concept demonstration of the reconfigurable micro-metastructures with a wide pattern-transformation spectrum for encryption application. **a**, Grayscale image of the *Mona Lisa* painting. **b**, Pixel-by-pixel mapping of the printed square metalattices composed of 10,000 cross units with different scanning powers, converted from the corresponding grayscale values. **c**, Schematics showing the pattern-transformation spectrum enabled by the printed cross units with different scanning powers. **d**, Optical images of a printed metalattice pattern with encoded scanning powers at 25 °C. Scale bar, 100 μm. The inset shows an enlarged image of the metalattice pattern (scale bar, 20 μm). **e**, Optical image showing the transformed patterns at 50 °C, and the emerging the *Mona Lisa*. Scale bar, 100 μm). **f**–**h**, Enlarged optical image showing the head (**f**), chest (**g**) and background (**h**) regions of the *Mona Lisa* painting. Scale bars in **f**, 40 μm and **g**,**h**, 20 μm. **i**, Solidity distribution of the enclosed regions formed by these neighbouring cross units before and after transformation. **j**, Aspect ratio distribution of the enclosed regions before and after transformation. **k**, Orientation of the close areas before and after transformation. a.u., arbitrary units. Source data

As a proof of concept, we were able to encode and map the grayscale information of any images in the printed structures for information encryption based on the wide range of reconfigurable patterns. As explained above, printing lattices with the same lattice size (that is, the same values of *D*) could be transformed into different patterns by continuously changing the scanning power or scanning speed during the 2PP process. As a demonstration, we converted the grayscale values of each pixel from a 100 × 100 pixel^2^ discretized image of the *Mona Lisa* painting (Fig. 5a) to a scanning power mapping of 10,000 printed cross units with an individual size of 10 × 10 µm^2^ (Fig. 5b) that adjacently interconnected without any gaps (Supplementary Fig. 14), enabling the programming of the metalattices. One typical feature of the LIHAM-actuated metastructures is their wide-spectrum programmability in the final patterns after transformation. As depicted in Fig. 5c, cross-composed lattices with different scanning powers could reversibly transform into different topologies on heating and cooling. Because the scanning powers were continuously addressed, the corresponding printed units could transform into wide-spectrum patterns.

Actuated by the LIHAM, the printed metalattice (Fig. 5d) emerged the image of the *Mona Lisa* painting as a collaborative result of these 10,000 buckled cross units at 50 °C (Fig. 5e). As predicted, the deformed patterns vary greatly in different locations and are easily distinguishable from each other. For example, the details in the head (Fig. 5f), chest (Fig. 5g) and background (Fig. 5h) regions of the painting are notably different. Besides, the metalattice actuated by the LIHAM shows good reversibility and stability, evidenced by the cyclic heating and cooling test (Supplementary Fig. 15). To better understand the transformation, we used some shape describers to quantitatively analyse the unit cells of the metalattices (Supplementary Fig. 16a). After the transformation, the area of the unit cells decreased along with the shrinkage of the LIHAM matrix (Supplementary Fig. 16b), and their distribution widened. Besides, the solidity (Fig. 5i) and circularity (Supplementary Fig. 16c) distributions shifted to low values and widened, indicating a high extent of shape transformation. The aspect ratio distribution of these unit cells also greatly widened after the transformation (Fig. 5j). Compared with the narrow distribution located around 1 (maximum 1.3) for the original metalattice pattern, the deformed aspect ratio distribution became much wider and the distribution shifted up to 4.7, proving the formation of a large number of new shapes. In addition, the defined orientation of these new patterns changed from the horizontal directions of the original lattices to other various directions (Fig. 5k), showing the diversity and complexity of the newly emerged patterns.

**Fig. 6:**
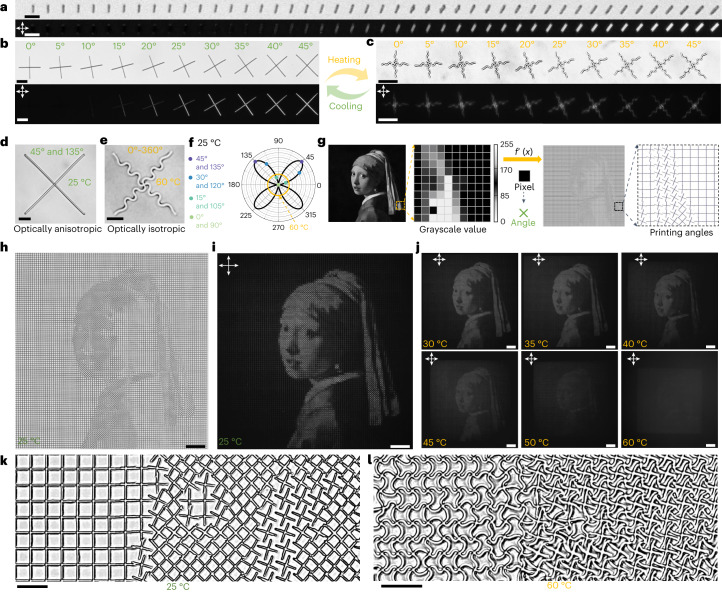
Proof-of-concept demonstration of the reconfigurable micro-metastructures with programmable optical anisotropy for encryption application. **a**, Optical and cross-polarized image sequentially showing a series of printed lines with angles ranging from 0° to 45°. Scale bar, 10 μm. **b**,**c**, Optical and cross-polarized image of a series of cross units with different orientations before (**b**) and after (**c**) actuation by the LIHAM. Scale bars, 25 μm. **d**,**e**, Optical images showing a 45° and 135° oriented cross before (**d**) and after (**e**) actuation. Scale bars, 10 μm. **f**, Light intensities distribution of the cross units before (black profile) and after (orange profile) actuation. **g**, Pixel-by-pixel mapping of the printed square metastructure composed of 10,000 cross units with different printing angle, converted from the corresponding grayscale values of the *Girl with a Pearl Earring* painting. **h**,**i**, Optical (**h**) and cross-polarized (**i**) image of the metastructures composed of the 10,000 cross units with different printing angles. Scale bars, 100 μm. **j**, Cross-polarized images showing the *Girl with a Pearl Earring* painting is gradually disappearing as the temperature increases. Scale bars, 100 μm. **k**,**l**, Enlarged optical images showing the pixelized metastructures before (**k**) and after (**l**) actuation. Scale bars, 20 μm. Source data

**Extended Data Fig. 3:**
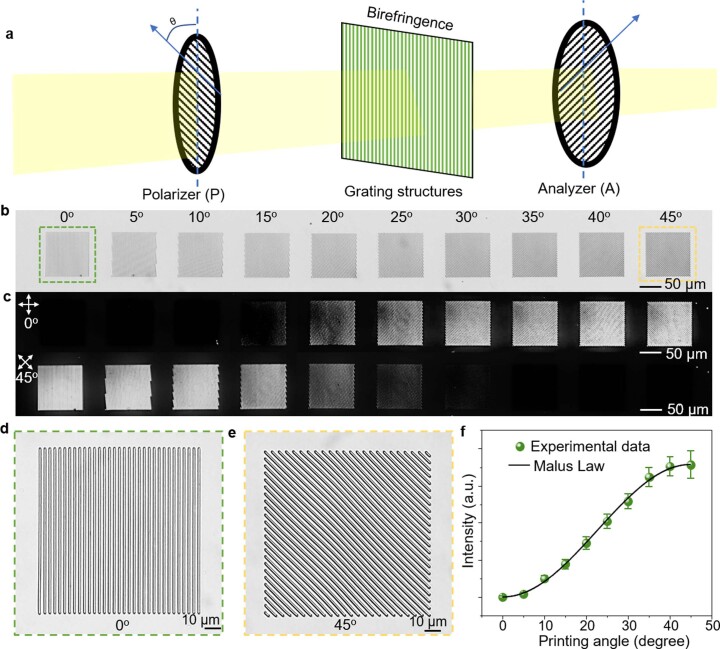
Birefringence behaviour of 2PP-printed grating structures. **a**, Schematic illustration showing a 2PP-printed grating observed under cross-polarizers. **b-c**, Optical (**b**) and cross-polarized (**c**) images of a series of squares composed of grating with different printed angles. **d-e**, Zoom-in optical images showing two squares composed of grating angle of 0° (**d**) and 45° (**e**), respectively. **f**, Light intensity of the squares measured under cross-polarizers as a function of the printed grating angles and the fitted curves according to Malus Law. Data points are shown as mean ± s.d. (n = 15). Source data

The wide-spectrum programmability of pixels presents exciting opportunities to incorporate additional properties into building blocks, endowing metastructures with more complex functionalities. Our research has revealed that 2PP-processed gratings exhibit birefringence behaviour under cross-polarizers due to the periodic variation in refractive index along the grating lines^40^ (for analysis, refer to Extended Data Fig. 3 and the Birefringence of the 2PP-processed polymer gratings section in the Methods). This angle-dependent birefringence behaviours could also be observed in the single printed beams (Fig. 6a) as well as two perpendicular beams (that is, cross units, Fig. 6b). Hence, we can leverage this polarization-dependent property to achieve additional anisotropic optical effects in individual cross units by providing them with different angle information. The cross units, when actuated by LIHAM, transform into two intersecting sinusoidal profiles, resulting in uniform light intensity under cross-polarizers (Fig. 6c), and the original optical anisotropy is eliminated. For instance, the buckling of a cross (45° and 135°_,_ Fig. 6d) after actuation endows the structures with omnidirectional optical information (0–360°, Fig. 6e). As depicted in Fig. 6f, the angle-dependent light intensity of various cross units (bold black profile) becomes angle-independent (orange circle) after actuation, and their initial angle information is lost.

**Extended Data Fig. 4:**
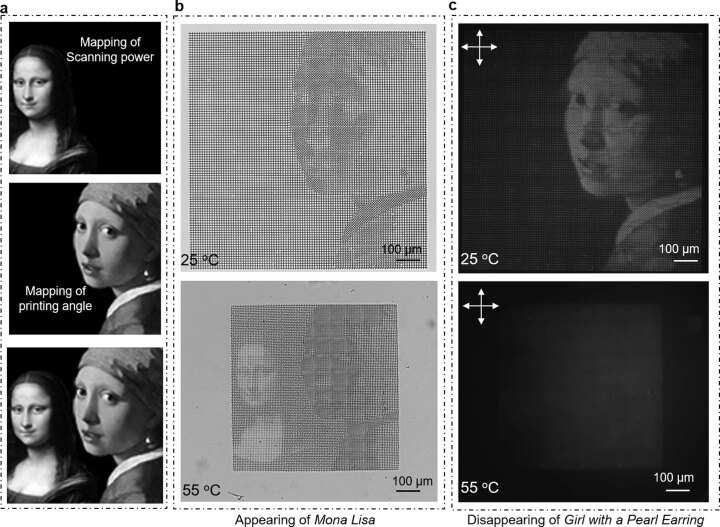
Integrated mapping of two images for complex encryption. **a**, Projection of the grayscale values of the *Mona Lisa* and *Girl with a Pearl Earring* images into an integrated mapping of scanning powers and printing angles. **b**, Optical image showing the appearing of the *Mona Lisa* image (mapping of scanning powers) upon heating. **c**, Cross-polarized image showing the disappearing of the *Girl with a Pearl Earring* image (mapping of printing angles) upon heating.

To reconstruct complex images such as the *Girl with a Pearl Earring* painting, we converted the grayscale values of each pixel from a discretized 100 × 100 pixel^2^ image into a printing angle mapping of 10,000 cross units with an individual size of 10 × 10 µm^2^ (Fig. 6g). The angle-dependent light intensity of each oriented cross-unit under cross-polarizers allowed us to reconstruct (Fig. 6h) and see the image under cross-polarizers (Fig. 6i). Notably, when actuated by the LIHAM, the encoded image in the metastructures gradually vanished as the rise of temperature (Fig. 6j), and could be fully recovered and reappear on cooling (Supplementary Video 9). The complex information hidden is achieved as the result of the structural buckling of the global cross units after actuation (Fig. 6k,l), which induces the switch between optical anisotropy to isotropy of the metastructures. Finally, we combined the pixelated information from two images and included an integrated mapping of scanning powers and printing angles (Extended Data Fig. 4a). When we heated the LIHAM, we were able to observe the *Mona Lisa* image appearing while the *Girl with a Pearl Earring* image disappeared (Extended Data Fig. 4b,c).

Overall, the linearly responsive hydrogel muscle-actuated reconfigurable 2D and 3D metamaterials with submicrometre precision demonstrate exceptional manipulation flexibility including global precise control over chirality transformation and site-specific manipulation of the wide pattern-transformation spectrum. The wide-spectrum programmability of our approach endows the basic building blocks of the metastructures with versatile transformations that collaboratively contribute to the reconstruction of intricate images with fine structures, as well as angle-dependent light-matter interactions for information hiding. These advanced capabilities offer unprecedented opportunities for constructing intricate and sophisticated information, suitable for various applications, including information cryptology, miniature robotics, photonics and phononics.

## Methods

### Synthesis of the various hydrogel muscles

All chemicals were used as received. Different temperature-responsive hydrogels were obtained by UV cross-linking (365 nm) their precursor solution for 15 min. Pure PNIPAM hydrogel precursor solution was obtained by vortex mixing *N*-isopropylacrylamide (NIPAM, monomer, Sigma-Aldrich, greater than or equal to 99%), *N*,*N*′-methylenebis(acrylamide) (BIS, cross-linker, Sigma-Aldrich, greater than or equal to 99%), Lithium phenyl-2,4,6-trimethylbenzoylphosphinate (TPO-Li, photoinitiator, Sigma-Aldrich, greater than or equal to 95%) and deionized water at the weight ratio of 500/4.7/3.7/5,000.

To develop large, transparent and uniformly deforming hydrogel, we adopted many strategies. For example, we used the interpenetrating polymer network strategy by in situ cross-linking the NIPAM monomer in the presence of another polymer network (polyvinyl alcohol, PVA). PNIPAM/PVA hydrogel precursor solution was prepared by vortex mixing NIPAM (monomer), BIS (cross-linker), TPO-Li (photoinitiator), PVA (5 wt%) solution (Sigma-Aldrich, average molecular weight 85,000–12,400) at the weight ratio of 500/4.7/3.7/5,000. The PVA solution is obtained by dissolving 5.23 g PVA into 100 ml of deionized water. The PNIPAM/PVA hydrogel shows a uniform and fast shrinkage, but it still becomes opaque (Supplementary Fig. 2 and Supplementary Video 2). We believe that the physical entanglements of the polymer chains were not strong enough to support and prevent the formation of light-scattering agglomerates.

We finally fabricate a LIHAM by adopting a terpolymerization strategy. LIHAM precursor solution was prepared by vortex mixing NIPAM (monomer), TPO-Li (photoinitiator), acrylamide (AM, monomer, Sigma-Aldrich, greater than or equal to 99%), 2-hydroxyethyl methacrylate (HEMA, monomer, Sigma-Aldrich, greater than or equal to 97%), polyethylene glycol diacrylate (PEGDA, cross-linker, Sigma-Aldrich, Mn 575), polyethylene glycol (PEG, a pore-forming agent; Sigma-Aldrich, Mn 400) and deionized water. To tailor the shrinkage and swelling abilities of the LIHAM, we explored the effects of the concentration of the monomers and their weight ratio. The molar concentration of the monomers, that is, NIPAM, HEMA and AM, determines the cross-linking density of the polymer network that affects the extent of desorption and/or absorption of water, allowing us to control the deformation degree of the hydrogels. As shown in Fig. 2f, LIHAM with a low molar concentration (0.69 M) of monomers shows a larger contraction (42%) than that with a higher concentration (2.76 M, 22%). The initial swelling of the LIHAM, when first absorbing water, also shows a similar trend. The weight ratio of three monomers (that is, NIPAM/HEMA/AM) can also determine the deformation of the LIHAM. The hydrophilic and hydrophobic transition will take place only in the responsive PNIPAM segments, while the other two passive polymer chains remain hydrophilic throughout the whole process. Therefore, the competition between the hydrophobic and hydrophilic (that is, responsive versus passive) polymer segments determines the deformation of LIHAM. As shown in Fig. 2g, higher ratios of the two hydrophilic chains reduce the contraction efficiency of LIHAM, simultaneously increasing the transition threshold temperatures and decreasing the maximum output strains. Unless otherwise stated, the LIHAM precursor solution contains a typical weight ratio of 500/10/29/29/150/300/4,500 (NIPAM/TPO-Li/AM/HEMA/PEGDA/PEG/deionized water).

### Photoresists for fabricating metastructures

To demonstrate our material-insensitive mechanism for reconfigurable metastructure, we used three photoresists with varying stiffness to fabricate micro-metastructures with different mechanical properties. For very soft metastructures (roughly 10 KPa), we prepared a photoresist by vortex mixing gelatin methacryloyl (Sigma-Aldrich, 80% degree of substitution), PEGDA (Sigma-Aldrich, Mn 250), TPO-Li and deionized water at a weight ratio of 115/25/25/1,000, followed by an ultrasonic treatment and filtration process (with a pore size of 200 nm). We used a commercial photoresist (DEGRAD INX N100, XPECT INX) for elastic metamaterials (roughly 50 MPa) and another commercial photoresist (IP-S, Nanoscribe GmbH) for rigid metamaterials (roughly 5.1 GPa). Unless otherwise stated, various metastructures were based on the DEGRAD INX N100 photoresist.

### Fabrication protocol for the reconfigurable metastructures

Extended Data Fig. 1 shows the detailed fabrication protocol for LIHAM-actuated reconfigurable micro-metastructures, which consists of three steps: (1) two-photon polymerization (2PP)-based micropatterning of the photoresist on a glass substrate (Nanoscribe, GmbH). It should be noted that automatic substrate calibration should be implemented before printing to ensure a uniform printing process and repeatable sample fabrication and actuation. After developing in different solvents (deionized water, acetone and 2-propanol for gelatin, DEGRAD INX N100 and IP-S photoresist, respectively), the metastructures on the glass substrate were fabricated. (2) A capillary cell was assembled above the metastructures using polystyrene balls coated with UV glue as the spacers. Large spacers (with a ball diameter of 214 µm, MicroParticles GmbH) are used for making thick LIHAMs to minimize the impact of metastructures on the deformation behaviours of LIHAMs. Then, the prepared hydrogel precursor was infiltrated into the capillary cell, followed by 15 min of UV exposure. (3) Once the cover glass of the capillary cell was removed, the LIHAM matrix with its embedded metastructures was sealed inside a polydimethylsiloxane chamber that was filled with deionized water. Subsequently, the actuation process was carried out within this aqueous environment. Before any measurement, the LIHAM matrix with embedded metastructures was heated to 50 °C, which helped it spontaneously detach and release from the glass substrate for further study (Supplementary Fig. 17).

### Characterization

An optical microscope (Zeiss; Axio Imager 2) observed with transmission mode was used to observe the transformation behaviours of metastructures. An atomic force microscope (NanoWizard 4, JPK Instruments) with a sharp tip (FIB3D2-100A, Bruker AFM probes) was used to measure the geometry of the printed structures. A scanning electron microscope (Leo Gemini 1530) was used to record the morphology of the printed 3D architecture. A confocal fluorescent microscope (SP8, Leica) was used to reconstruct the 3D architecture before and after heating. A rheometer (Discovery HB20; TA Instruments) was used to measure the mechanical properties of LIHAM at different temperatures. Before measuring the shape-morphing behaviours of various hydrogel muscles, a laser cutter (ProtoLaser, LPKF Laser & Electronics AG) was used to cut the hydrogel muscles into squares with a side length of 1.5 mm. The geometry parameters for beams and grids are determined by analysing recorded optical images and AFM results.

### Shape description of the unit cells

To better understand how the shapes of the unit cells of the metastructures evolve, some shape descriptors were used. Circularity is defined by 4*π* × area/perimeter^2^, which describes how close a unit cell is similar to a perfect circle: a value of 1 indicates it is a perfect circle; values approaching 0 indicate an elongated shape that deviates from a perfect circle. Aspect ratio (greater than or equal to 1) is defined by the length ratio of the major axis and the minor axis of the fitted ellipse, which describes the general form of a unit cell. Orientation (0–180°) is the angle between the major axis and *x* axis of the unit cell. Solidity is defined by the area ratio of a unit cell and the convex hull, which is the measurement of the overall concavity of a unit cell.

We monitored the evolution of aspect ratio and solidity of the *λ*-structured and *λ*/2-structured metalattices. Before reaching the threshold temperature for buckling, the aspect ratio and solidity of the unit cells of the two lattices remained around 1 (Fig. 4m,n), indicating that no geometric transformation could be observed with only uniform shrinkage in size. After reaching the threshold temperatures (38 °C for the *λ*-structured metalattice and 43 °C for the *λ*/2-structured metalattice), a snap buckling of the constituent beams resulted in a dramatic geometrical difference and their solidity decreased. The unit cells of the *λ*-structured metalattices always remained rotationally symmetrical, and thus maintained their aspect ratio as 1, while the aspect ratio for the *λ*/2-structured metalattices increased to 4.5 because of their gradual elongation.

### Birefringence of the 2PP-processed polymer gratings

The DEGRAD INX N100 photoresist is used as the photoresist for creating 2PP-processed polymer gratings. These gratings are printed in arrays of squares with various angles ranging from 0–45°. The printed arrays have a length of 100 µm, a thickness of 500 nm and a grating period distance of 3 µm. The experimental setup is shown in Extended Data Fig. 3a, and the printed gratings show birefringence behaviours under cross-polarizers (Extended Data Fig. 3b,c). When aligned with their length parallel to the direction of the polarizer (or vertical to the analyser), the printed arrays of squares with a printing angle of 0° (Extended Data Fig. 3d) appear in a completely dark field of view (extinction mode). As the printing angle increases to 45° (Extended Data Fig. 3e), the light intensity gradually increases to its maximum brightness. When a rotation of 45° is applied to these squares, their light intensities exchange in the opposite way.

As the printing angles increase from 0° to 45°, the light intensity distribution of these squares under cross-polarizers is in accordance with the Malus Law (Extended Data Fig. 3f). The 2PP-processed polymer gratings have varying refractive indices along the direction of the grating lines, which causes the incident light to split into two polarized components. One component is parallel to the polymer grating lines, while the other is perpendicular to them. These two polarized components experience different amounts of diffraction as they pass through the polymer grating. As a result, the diffracted beams of light from the polymer grating have different polarization directions and different diffraction angles, which can be observed when the polymer grating is viewed under cross-polarizers. The printed single polymer line also shows birefringence behaviours due to the different refractive indices of the line along the two directions, which can be used as the building blocks to encode complex information inside metastructures.

### FEA

FEA was performed using commercial software (FE software, ABAQUS/Standard, v.2020, Dassault Systèmes). To understand the transformation behaviours of the printed beams. To simplify the computational complexity of the FEA, 2D plane strain conditions were assumed and the model was constructed with CPEG8 elements. Generalized plane strain conditions were used to prevent stress generation due to thermal expansion and/or contraction in the out-of-plane direction. The analysis model consisted of a beam of size 150 × 1 um placed in the centre of a rectangular slab of hydrogel (250 × 40 um). The large size of the hydrogel is necessary to prevent boundary effects in the buckling of the beam. The mesh was generated in a manner such that there were at least eight elements along the thickness of the beams and at least 80 elements in one wavelength of the buckled geometry, to accurately capture the nonlinear buckling of the beams. Both the printed material and the hydrogel were modelled as linear elastic materials, with the hydrogel having a temperature-dependent elastic modulus (Supplementary Fig. 18). Thermal contraction of the hydrogel was prescribed by using the thermal expansion ratio found experimentally. The simulations included an initial buckling analysis to obtain the buckling mode of the structure, followed by a nonlinear postbuckling analysis (dynamic implicit step), where a perturbation in the form of the critical buckling mode was introduced in the mesh to study the large deformation, postbuckling response. Quasistatic conditions were ensured during the postbuckling step by monitoring the kinetic energy and introducing a small damping factor.

### Analysis of the LIHAM-actuated shape-morphing behaviours

To understand the buckling-induced in-plane transformation of reconfigurable metastructures, an analytical model of a basic unit (a beam with a rectangular cross-section) was used based on surface elastic theory. The total potential energy of the system includes three components: the bending (*U*_b_) and membrane energy (*U*_m_) of the printed beam and the strain energy (*U*_h_) of the LIHAM matrix. The profile of the deformed beam can be described by *y* = *A*cos*kx*, the bending energy per unit length (*U*_b_) of the deformed beams is therefore:

1 $${U}_{\mathrm{b}}=\frac{k}{2\uppi }{\int }_{0}^{\frac{2\uppi }{k}}\frac{{E}_{\mathrm{p}}I}{2}{\left(\frac{{\mathrm{d}}^{2}y}{{\mathrm{d}}{x}^{2}}\right)}^{2}{{\mathrm{d}}x}=\frac{{E}_{\mathrm{p}}I}{4}{A}^{2}{k}^{4}$$

where *E*_p_ is the Young’s modulus of the printed beam and *E*_p_*I* is its bending stiffness.

For membrane strain (*ε*_m_), it is defined by normal deflection (*y*) and axial displacement (*u*) along the beam axis $\left({\varepsilon }_{\mathrm{m}}=\frac{{{\mathrm{d}}y}}{{{\mathrm{d}}x}}+\frac{1}{2}{\left(\frac{{{\mathrm{d}}u}}{{{\mathrm{d}}x}}\right)}^{\!{2}}\right)$. Shear stress at the interface between the deformed beam (roughly 50 MPa) and the LIHAM matrix (roughly 20 KPa) is negligible due to their large difference in Young’s modulus. A constant membrane strain is generated as a result of force equilibrium in the deformed beams, which enables axial displacement $u=\frac{{{k}}{A}^{2}{{\sin }}\left(2{kx}\right)}{8}-{\varepsilon }_{\mathrm{LIHAM}}x$, where *ε*_LIHAM_ is the deformation strain at a certain temperature. The membrane strain is therefore ${\varepsilon }_{\mathrm{m}}=\frac{{k}^{2}{A}^{2}}{4}-{\varepsilon }_{\mathrm{LIHAM}}$, and the membrane energy can be obtained:

2 $${U}_{\mathrm{m}}=\frac{k}{2\uppi }{\int }_{0}^{\frac{2\uppi }{k}}\frac{{E}_{\mathrm{p}}R}{2}{{\varepsilon }_{\mathrm{m}}}^{2}{{\mathrm{d}}x}=\frac{{E}_{\mathrm{p}}R}{2}{\left(\frac{{k}^{2}{A}^{2}}{4}-{\varepsilon }_{\mathrm{LIHAM}}\right)}^{2},$$

where *R* is the area of the cross-section of the printed beam.

The normal force applied to the printed beam due to deformation can be used to obtain the strain energy of the LIHAM matrix. The thickness (214 µm) of the LIHAM matrix is much larger than that of the printed beam (roughly 1 µm), so the LIHAM matrix can be regarded as a semi-infinite solid. On the basis of beam theory, we can obtain the applied normal force (*F*) on the printed beam due to deformation as $F=\frac{{E}_{\mathrm{p}}I{\mathrm{d}}^{4}y}{{\mathrm{d}}{x}^{4}}-\frac{{E}_{\mathrm{p}}R{\varepsilon }_{\mathrm{m}}{\mathrm{d}}^{2}y}{{\mathrm{d}}{x}^{2}}=-{P{\mathrm{cos}}kx}$, where $P=-{E}_{\mathrm{p}}{RA}{y}^{2}\left(\frac{{k}^{2}{A}^{2}}{4}-{\varepsilon }_{\mathrm{LIHAM}}\right)-{E}_{\mathrm{p}}I{k}^{4}$, so the LIHAM matrix bears a lateral traction (*P*cos*kx*), which causes a normal deflection (*d*) of a point (*x*, *y*_1_) on the surface to the centre of the printed beam as

$$\begin{array}{l}d={P{\mathrm{cos}}kx}\left\{l\left[(1-\nu )^{-1}+{\mathrm{ln}}2-\gamma \right]-\left(\frac{l}{2}+y\right){\rm{ln}}\left(k\left|\frac{l}{2}+{y}_{1}\right|\right)\right.\\\qquad\left.-\left(\frac{l}{2}-{y}_{1}\right){\rm{ln}}\left(k\left|\frac{l}{2}-{y}_{1}\right|\right)\right\}/\left(\frac{\pi {E}_{\mathrm{m}}^{{\prime} }l}{2}\right),\end{array}$$

where *ν* is the Poisson’s ratio of the LIHAM matrix, *γ* is Euler’s constant (0.577), *l* is the length at the cross-section of the printed beam and ${E}_{\mathrm{m}}^{{\prime} }={E}_{\mathrm{m}}/(1-{\nu }^{2})$ is the plain strain modulus of the LIHAM matrix (*E*_m_ is Young’s modulus of the LIHAM matrix). On the basis of the divergence theorem, the strain energy per unit length (*U*_h_) can be obtained as:

3 $${U}_{\mathrm{h}}=\frac{k}{2\uppi }{\int }_{0}^{\frac{2\pi }{k}}{\int }_{-\frac{l}{2}}^{\frac{l}{2}}\frac{1}{2}\frac{{P{\mathrm{cos}}kx}}{l}{d{\mathrm{d}}}x{\mathrm{d}}{y}_{1}=\frac{{P}^{2}}{4\uppi {E}_{\mathrm{m}}^{{\prime} }}\left(\frac{3-\nu }{1-\nu }-2\gamma -2\mathrm{ln}\frac{{kl}}{2}\right).$$

Therefore, the total potential energy (*U*_all_, per unit length) in the system is:

4 $$\begin{array}{l}{U}_{\mathrm{all}}={U}_{\mathrm{b}}+{U}_{\mathrm{m}}+{U}_{\mathrm{h}}-\frac{k}{2\uppi }{\displaystyle\int }_{0}^{\frac{2\uppi }{k}}{\displaystyle\int }_{-\frac{l}{2}}^{\frac{l}{2}}\frac{{P{\mathrm{cos}}kx}}{l}\left(d-y\right){\mathrm{d}}x{\mathrm{d}}{y}_{1}\\ \qquad=\frac{{E}_{\mathrm{p}}I}{4}{A}^{2}{k}^{4}+\frac{{E}_{\mathrm{p}}R}{2}{\left(\frac{{k}^{2}{A}^{2}}{4}-{\varepsilon }_{\mathrm{LIHAM}}\right)}^{2}+\frac{1}{2}{PA}-\frac{{P}^{2}}{4\uppi {E}_{\mathrm{m}}^{{\prime} }}\left(\frac{3-\nu }{1-\nu }-2\gamma -2\mathrm{ln}\frac{{kl}}{2}\right),\end{array}$$

where the last part: $\frac{k}{2\uppi }{\int }_{0}^{\frac{2\uppi }{k}}{\int }_{-\frac{l}{2}}^{\frac{l}{2}}\frac{{P{\mathrm{cos}}kx}}{l}\left(d-y\right){\mathrm{d}}x{\mathrm{d}}{y}_{1}$ is the work at the interface between the printed beam and the LIHAM matrix due to the traction-induced mismatch. By minimization of the total potential energy for the in-plane buckling system (with regard to *A* and *k*), we can obtain the theoretical solutions of their characteristic geometries (wavelength and amplitude of the deformed beams):

5 $${\left(\frac{{E}_{\mathrm{p}}I}{{E}_{\mathrm{m}}^{{\prime} }}\right)}^{\!\frac{1}{4}}k={\left[\frac{2\uppi \left(\frac{1}{1-\nu }-\gamma -{\rm{ln}}\frac{{kl}}{2}\right)}{{\left(\frac{3-\nu }{1-\nu }-2\gamma -2{\rm{ln}}\frac{{kl}}{2}\right)}^{2}}\right]}^{1/4}$$

6 $$A=\frac{2}{k}\sqrt{{\varepsilon }_{\mathrm{LIHAM}}-{\varepsilon }_{0}}$$

where ${\varepsilon }_{0}=\{{EI}{k}^{4}+\uppi {E}_{\mathrm{m}}^{{\prime} }{\left[(3-\nu ){(1-\nu )}^{-1}-2\gamma -2{\rm{ln}}\frac{{kl}}{2}\right]}^{-1}/\left({ER}{k}^{2}\right)\}$ is a threshold strain of the LIHAM matrix, and sinusoidal structures become appreciable after reaching the critical actuation strain (Fig. 3f, stage II). Before that, only uniform compression to the printed beam can be found (Fig. 3f, stage I). The right part of equation (5) can be regarded as a constant due to a quarter of the power functions changing very slowly with *kl*, and at a certain temperature, $\sqrt{{\varepsilon }_{\mathrm{LIHAM}}-{\varepsilon }_{0}}$ can be a constant, so the geometries of the transformed beams can be determined by:

7 $$\lambda =m{\left(\frac{{E}_{\mathrm{p}}I}{{E}_{\mathrm{m}}^{{\prime} }}\right)}^{\frac{1}{4}}={\mathrm{m}}^{{\prime} }{({E}_{\mathrm{p}}h{l}^{3})}^{\frac{1}{4}}$$

8 $$A=n{\left(\frac{{E}_{\mathrm{p}}I}{{E}_{\mathrm{m}}^{{\prime} }}\right)}^{\frac{1}{4}}={n}^{{\prime} }{({E}_{\mathrm{p}}h{l}^{3})}^{\frac{1}{4}}$$

where *m*, *m*′, *n* and *n*′ are different constants. So, their characteristic geometries (*A* and *λ*) at a certain temperature can be predicted if we know the cross-section geometries (*l* and *h*) of the printed beams (measured by AFM), which can be programmed by applying different scanning powers during the 2PP micropatterning process. As shown in Fig. 3k, good linear relationships (*R*^2^ = 0.994 for *λ*, and *R*^2^ = 0.977 for *A*) can be obtained, demonstrating the validity of the model. From the relationship, we can understand the transformation behaviour strongly depends on the cross-section geometries, while the mechanical properties (*E*_p_) of the printed beams have a smaller impact on their transformation behaviours, which was evidenced by the successful actuation of printed beams, such as both soft and rigid printed beams based on gelatin photoresist and IP-S photoresist. Therefore, this transformation strategy using hydrogel muscles to actuate metastructures can be applied to various printable photoresists and other patterning technologies for metastructure at different scales.

Besides the in-surface buckling mode, we also observed different buckling modes (out-of-plane buckling, Supplementary Fig. 19a–e), during the actuation of printed beams (Supplementary Fig. 19b) and the composed metalattice (Supplementary Fig. 19d). The analysis of total potential energy for different buckling modes is similar, with the only difference being the area moment of inertia of the printed beam along different directions (out-of-plane and in-plane). The competition between out-of-plane and in-plane buckling can be determined by the cross-sectional geometry of the printed beams, which can be controlled by adjusting the laser dosage (scanning powers or scanning speeds). In the work, we focus on the in-plane buckling mode of the metastructures, which are printed with scanning powers higher than 10 mW.

## Online content

Any methods, additional references, Nature Portfolio reporting summaries, source data, extended data, supplementary information, acknowledgements, peer review information; details of author contributions and competing interests; and statements of data and code availability are available at 10.1038/s41563-023-01649-3.

### Supplementary information

Supplementary Information Supplementary Figs. 1–19.

Supplementary Video 1 Deformation of a conventional PNIPAM hydrogel during heating and cooling.

Supplementary Video 2 Deformation of a PNIPAM/PVA hydrogel during heating and cooling.

Supplementary Video 3 Deformation of a LIHAM during heating and cooling.

Supplementary Video 4 Buckling and recovery of a beam when heating and cooling.

Supplementary Video 5 Achiral transformation of a square metalattice (printed with 50 mW) with a hatching distance of 10 µm.

Supplementary Video 6 Chiral transformation of a square metalattice (printed with 20 mW) with a hatching distance of 10 µm.

Supplementary Video 7 Geometrical transformation of a triangular metalattice (printed with 30 mW) with a hatching distance of 10 µm.

Supplementary Video 8 3D reconstructed rotation showing geometrical transformations of a tent-like 3D architecture (IP-S).

Supplementary Video 9 Disappearing (on heating) and recovery (on cooling) of the *Girl with a Pearl Earring* image in metastructure (150 × 180 pixels^2^, 27,000 units) actuated by the LIHAM.

Supplementary Data.

### Source data

Source Data Fig. 2 Statistical source data.

Source Data Fig. 3 Statistical source data.

Source Data Fig. 4 Statistical source data.

Source Data Fig. 5 Statistical source data.

Source Data Fig. 6 Statistical source data.

Source Data Extended Data Fig. 3 Statistical source data.

## Data Availability

Source data are provided with this paper. The data needed to evaluate the conclusions in this work are publicly available online. Additional data related to this paper may be requested from the corresponding authors upon reasonable request.
