# Supplementary material for: Hydrogel muscles powering reconfigurable micro-metastructures with wide-spectrum programmability
Source: Nat Mater. Author manuscript; Available in PMC 2023 Oct 5. (PMC10533409; doi:10.1038/s41563-023-01649-3)
Supplement: Supplementary Information [file EMS185289-supplement-Supplementary_Information.pdf]

nature materials

Article https://doi.org/10.1038/s41563-023-01649-3

Hydrogel muscles powering reconfigurable micro-metastructures with wide-spectrum programmability

In the format provided by the
authors and unedited

This PDF file includes:

Supplementary Figs. 1 to 19
Captions for Supplementary Videos 1 to 9

Other Supplementary Materials for this manuscript include the following:

Supplementary Videos 1 to 9

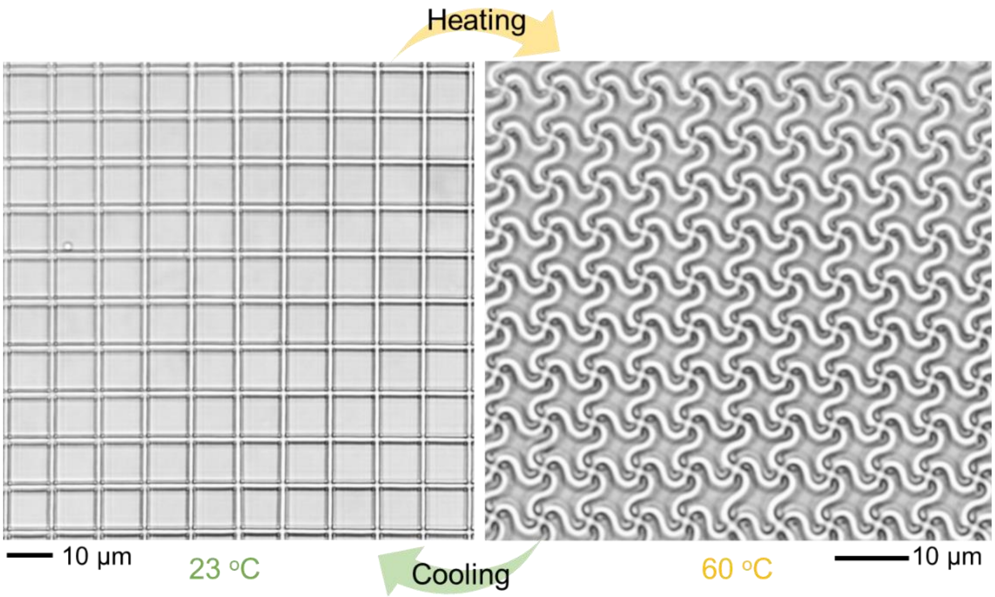

Supplementary Fig. 1 A 3D-printed meta-lattice could globally transform into a new uniform pattern (a one-wavelength structure between two neighbouring nodes) upon heating and revert to the original lattice when cooling.

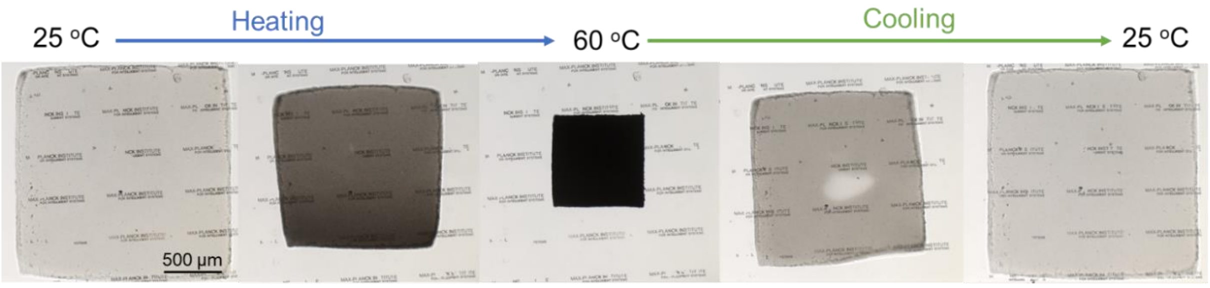

Supplementary Fig. 2 Photo snapshots showing the shape evolution of the PNIPAM/PVA hydrogel.

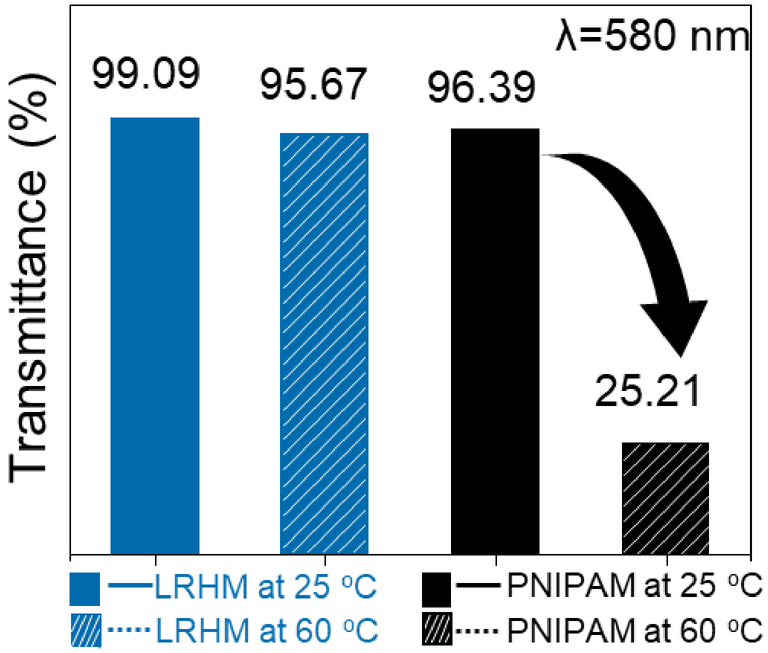

Supplementary Fig. 3 Comparison showing the transmittance (at the wavelength of 580 nm) of LIHAM and PNIPAM hydrogels at 25 °C and 60 °C.

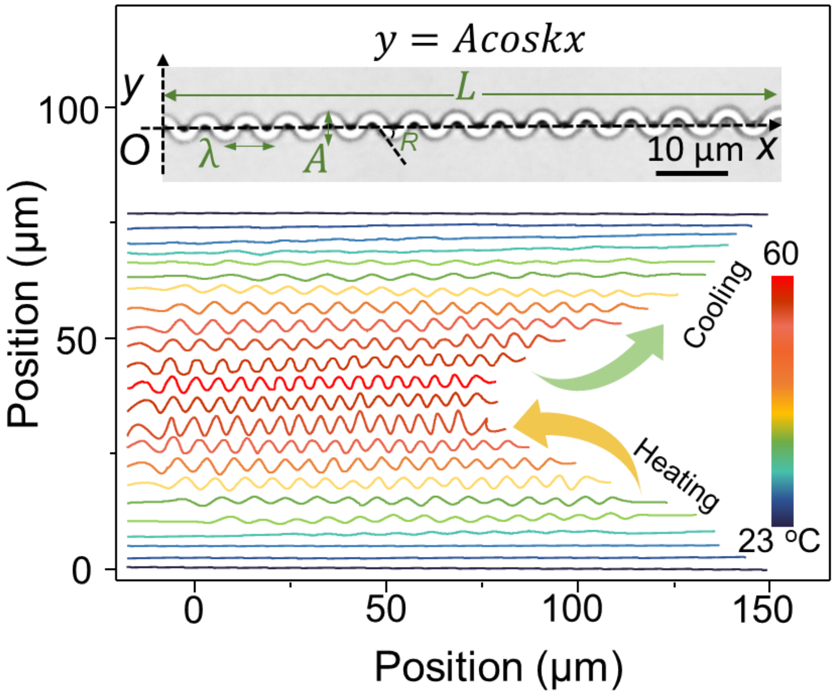

Supplementary Fig. 4 Experimental results tracking the evolution of topological profiles of a buckling beam (15 mW) at different temperatures.

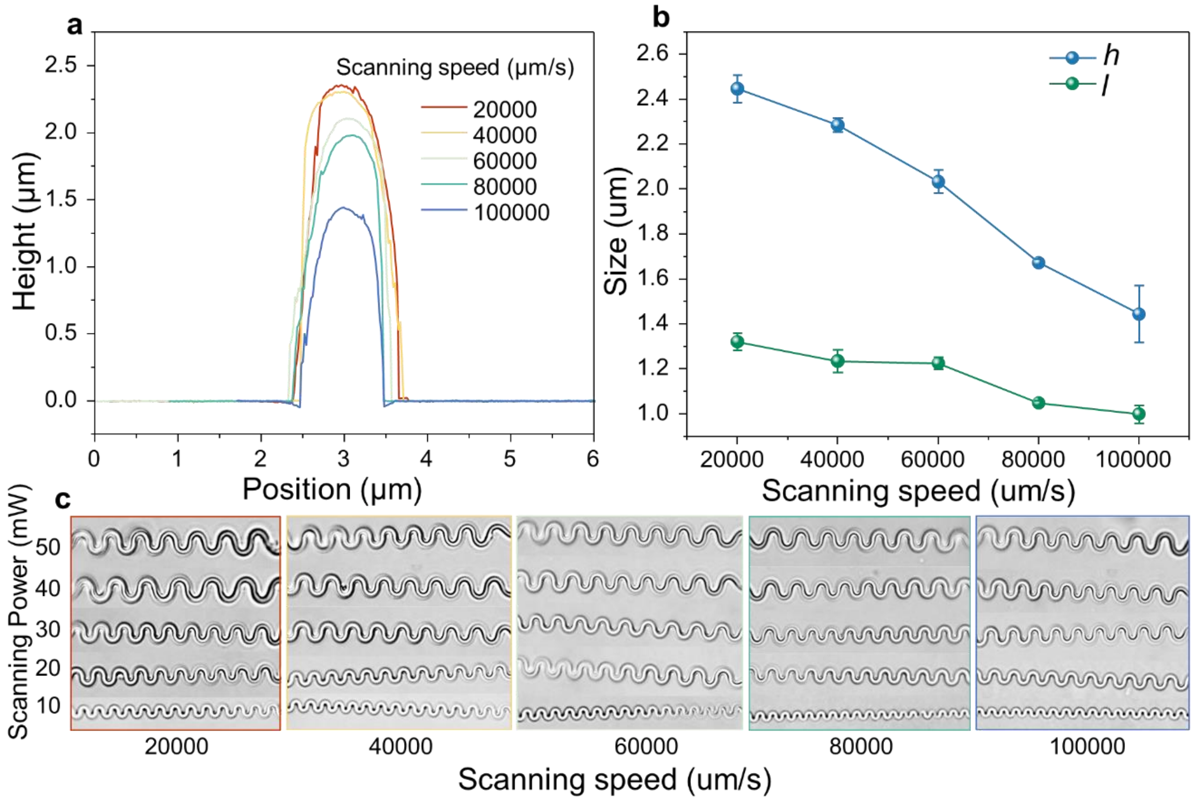

Supplementary Fig. 5 Effect of scanning speed on the cross-section profiles and shape-morphing behaviors. a, Cross-section profiles of the printed beams with different scanning speeds. **b**, Height (*h*) and length (*l*) of the cross-sections of the printed beams with different scanning speeds. Data points are shown as mean ± s.d. (n=18). **c**, Optical microscope images showing morphologies of the transformed beams at 60 °C under different scanning powers and scanning speeds.

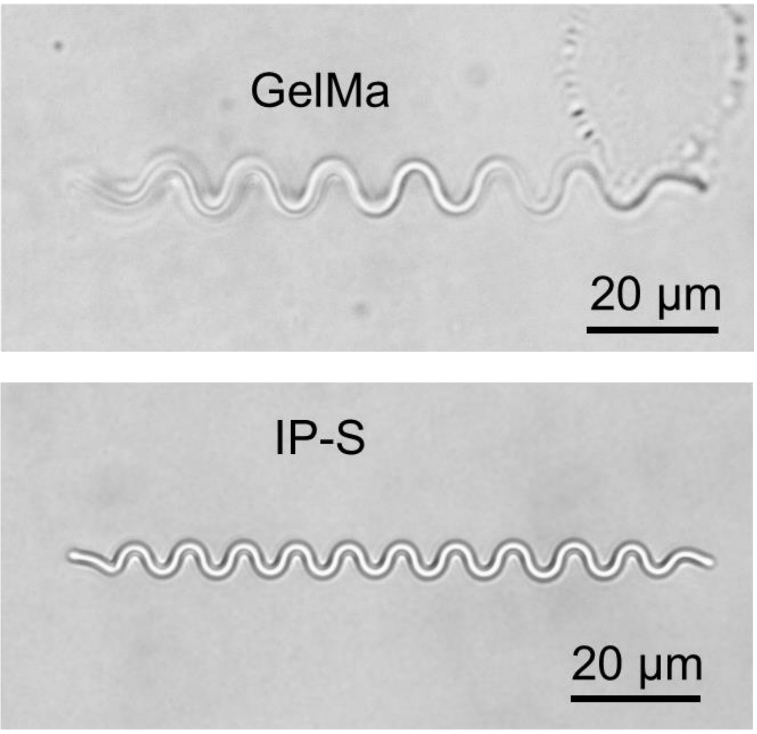

Supplementary Fig. 6 Optical images of two buckled beams fabricated using GelMa and IP-S photoresists respectively, demonstrating the material-insensitive mechanism.

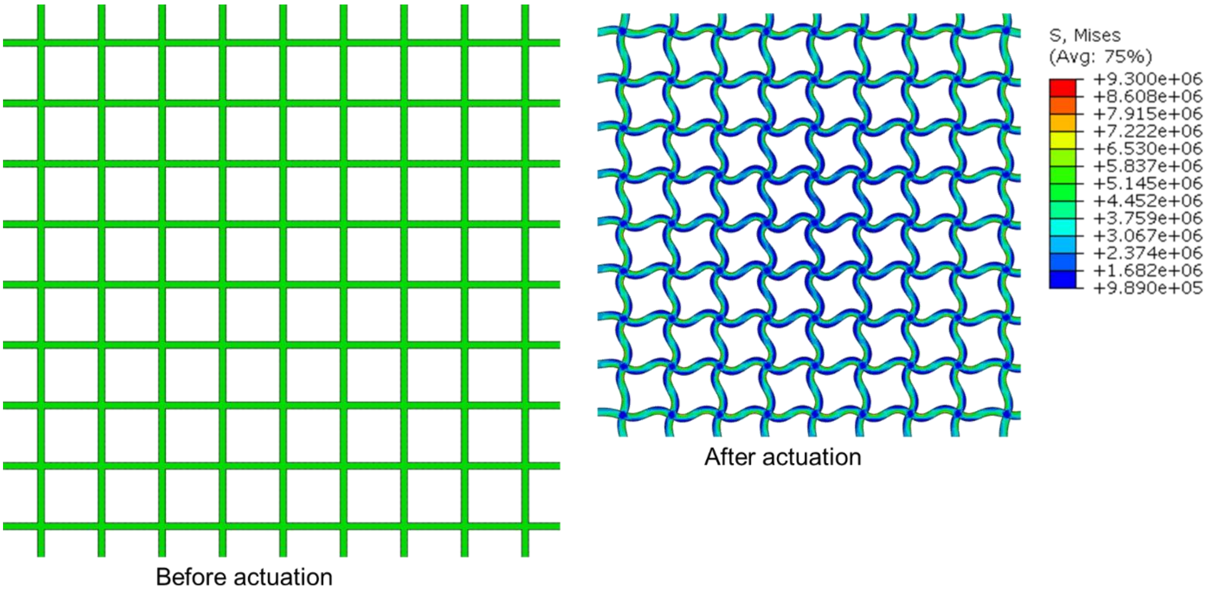

Supplementary Fig. 7 A finite element analysis (FEA) result showing a 2D grid before and after actuation, where one-wavelength between two adjacent nodes is shown in this case.

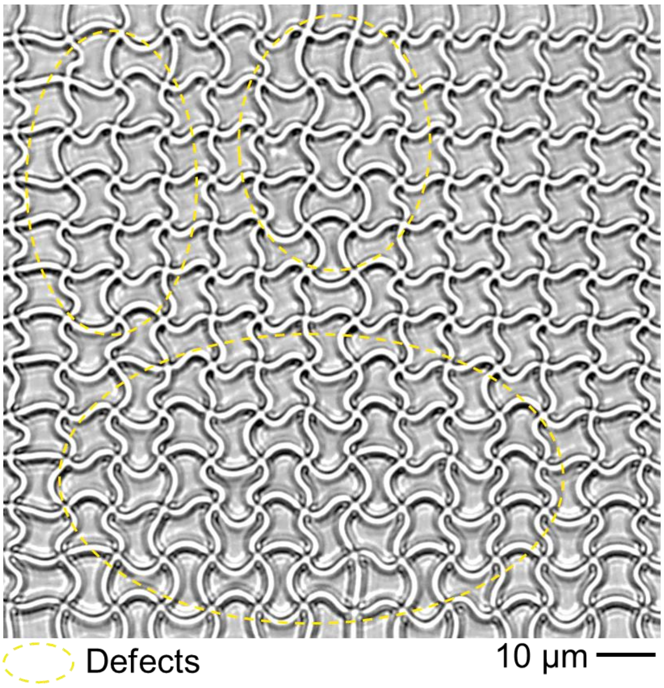

Supplementary Fig. 8 Optical image showing the generation of a structural failure from a printed lattice that does not meet the design principle ($D = \frac{n}{2}\lambda$) after transformation at 50 ^{o}C.

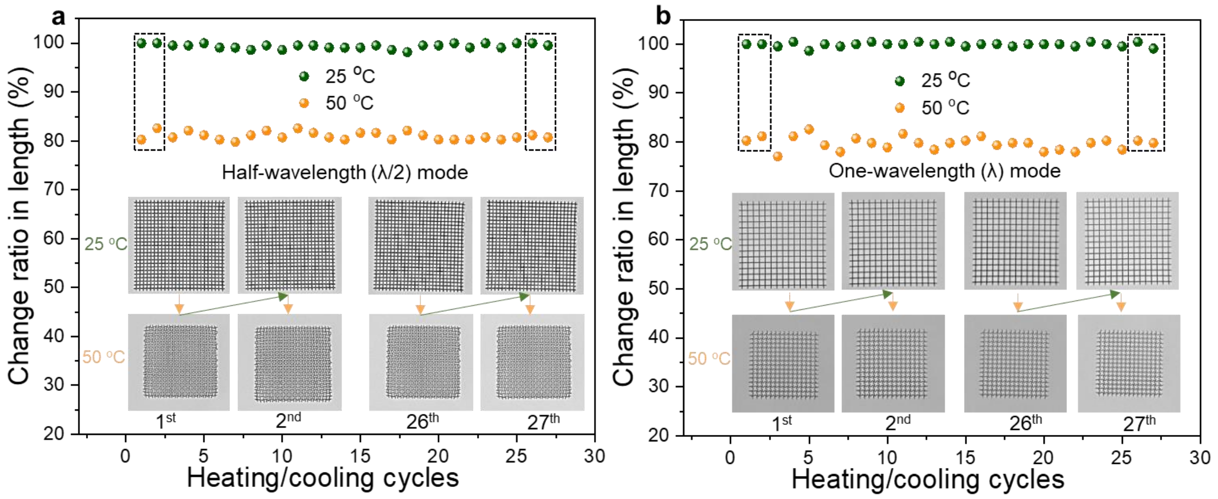

Supplementary Fig. 9 Cyclic actuation of two kind of grids. a-b, Cyclic heating/cooling actuation tests for two transformation modes with half-wavelength (λ/2) (**a**) and one wavelength (λ) (**b**).

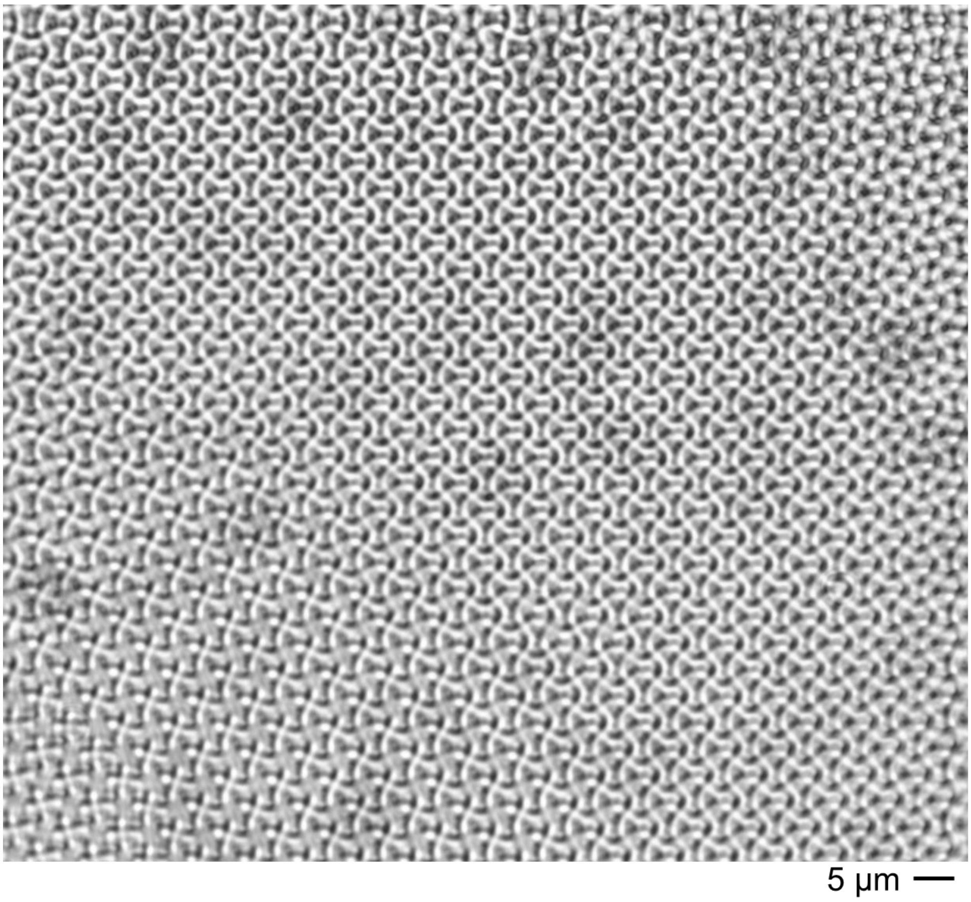

Supplementary Fig. 10 Optical image showing a metastructure (200×200 μm^2) with a unit density of ten million per square centimeter.

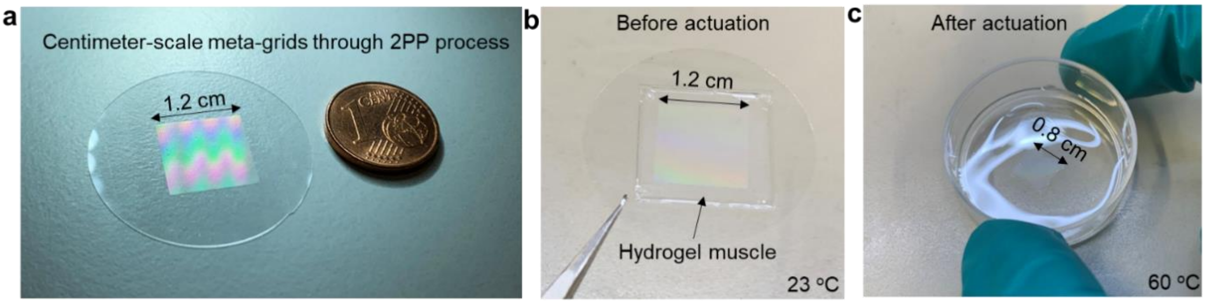

Supplementary Fig. 11 Centimeter-scale reconfigurable metastructures fabricated by the 2PP process. **a**, Photograph showing a 2PP-prined meta-lattice (1.2 × 1.2 cm²) with a grid period of 10 µm. **b**, Photograph showing the meta-lattice inside the hydrogel muscle before actuation. **c**, Photograph showing the shrinkage of the meta-lattice after actuation.

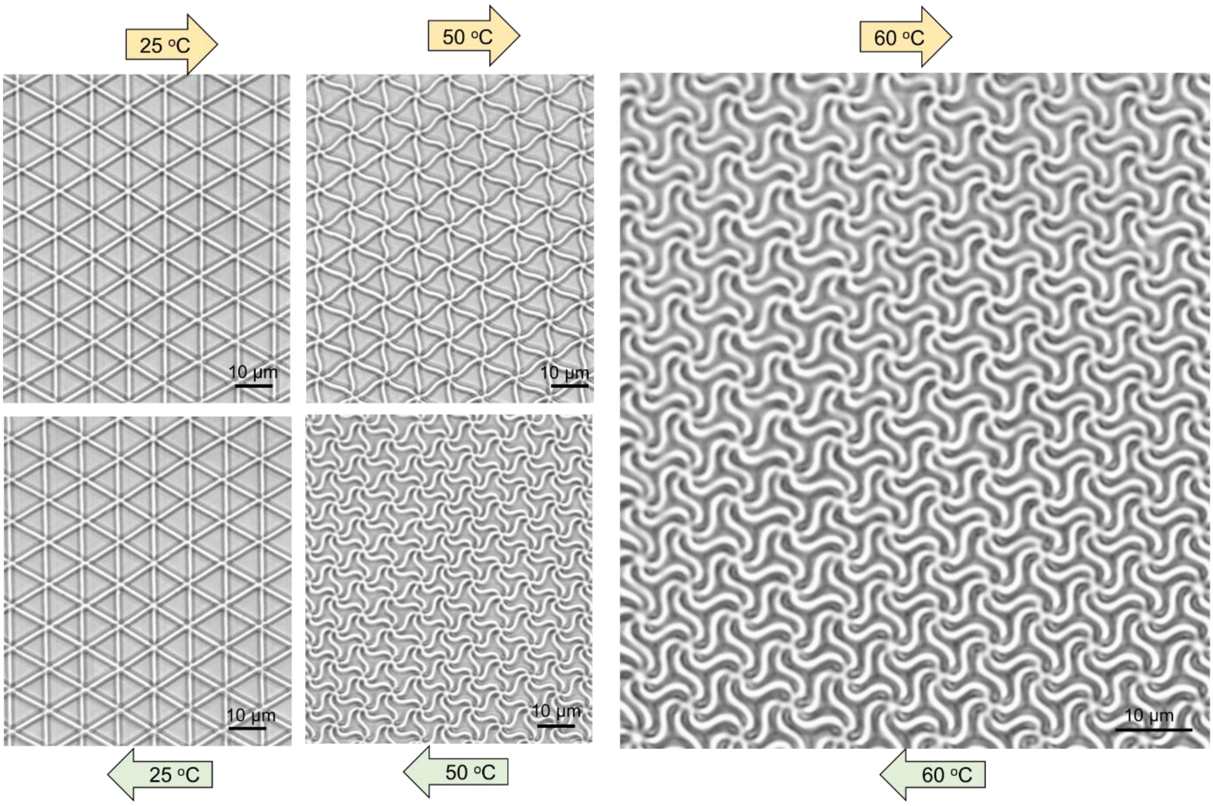

Supplementary Fig. 12 Optical images showing the transformation evolution of a triangular lattice metastructure at different temperatures.

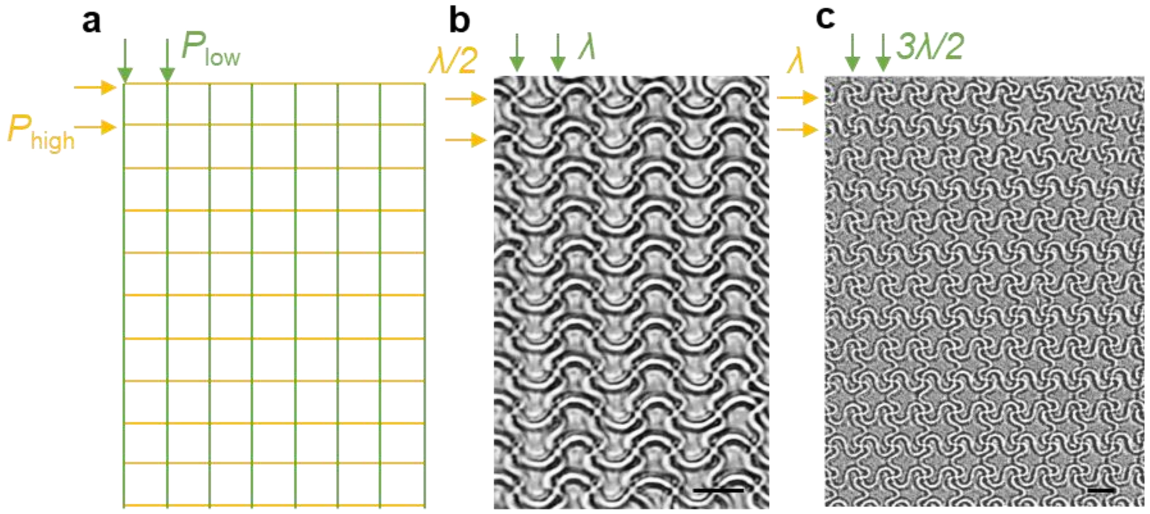

Supplementary Fig. 13 Inhomogeneous printing of meta-grids and their topological transformations. a, Design of inhomogeneous meta-grids with different scanning powers along different directions. **b**, Optical images showing a pattern with half-wavelength (λ/2) structures between two horizontal nodes and one-wavelength (λ) structures between two vertical modes. The scale bar is 10 µm. **c**, Optical images showing a pattern with one-wavelength (λ) structures between two horizontal nodes and one-and-half-wavelength (3λ/2) structures between two vertical modes. The scale bar is 10 µm.

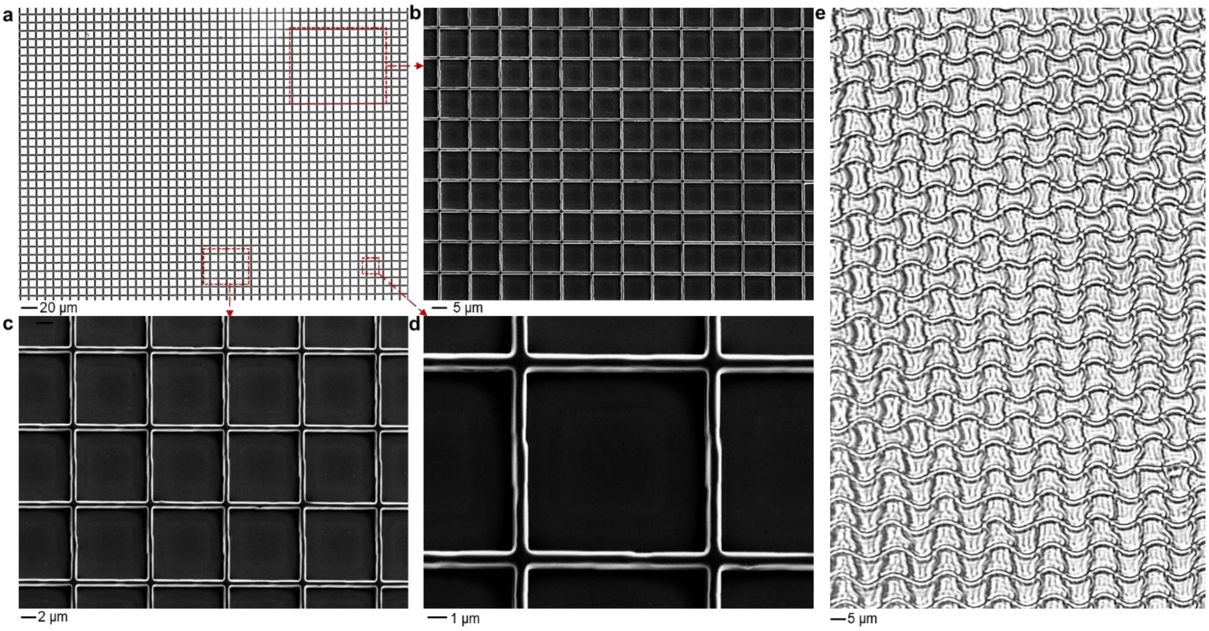

Supplementary Fig. 14 Morphologies of a meta-lattice before and after actuation. **a**, Optical image of a printed pixelized lattice. **b-d,** SEM image of the printed pixelized lattice under different magnifications. **e,** Optical image of the actuated lattice. No disconnections can be observed among these cross units of the meta-lattice before and after actuation.

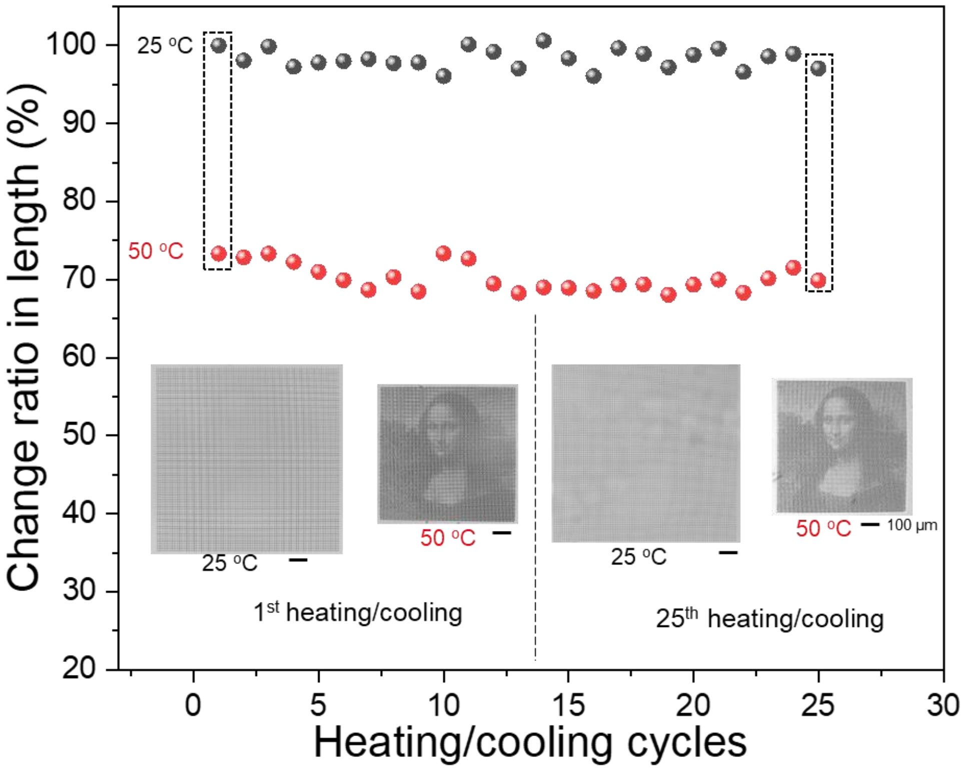

Supplementary Fig. 15 Cyclic heating and cooling actuation for the meta-lattice composed of 10000 units. Insets are the optical images showing 1st and 25th cyclic heating and cooling.

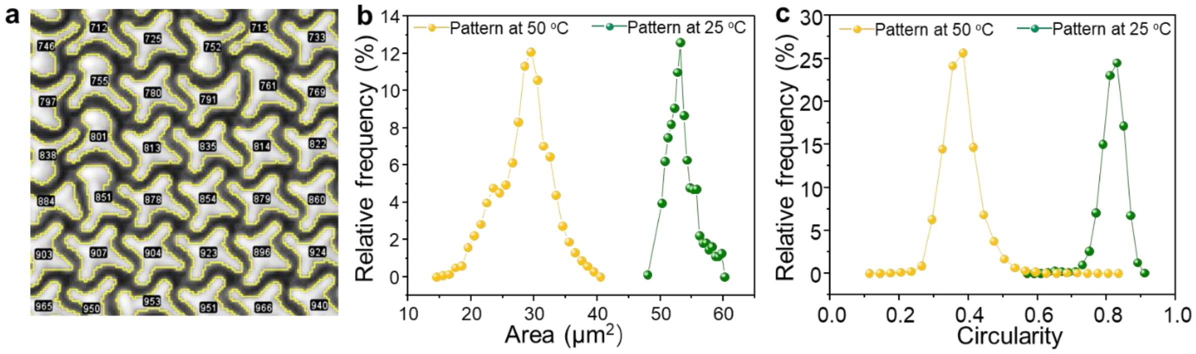

Supplementary Fig. 16 Image processing of the 10000-unit composed metastructures before and after heating. a, A snapshot showing the geometrical analysis of deformed unit cells. **b**, Area distribution of the enclosed regions formed by these neighboring cross units before and after transformation. **c**, Circularity distribution of the enclosed regions formed by these neighboring cross units before and after transformation.

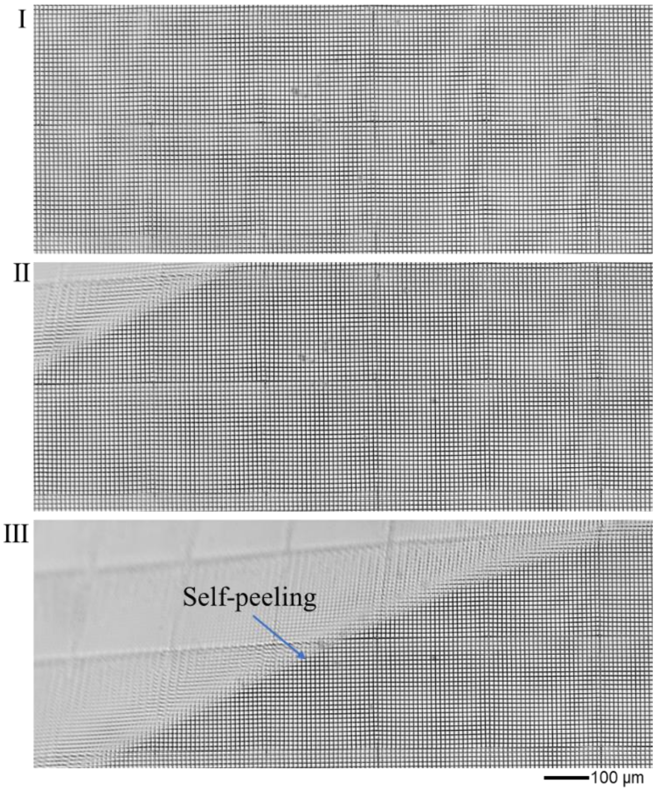

Supplementary Fig. 17 Optical images sequentially showing the self-peeling of the metastructures (1.2×1.2 cm^2) on the glass substrate at 50 ºC, which should be conducted before any further experiments to release the metastructure-embedded LIHAM matrix fixed from substrate.

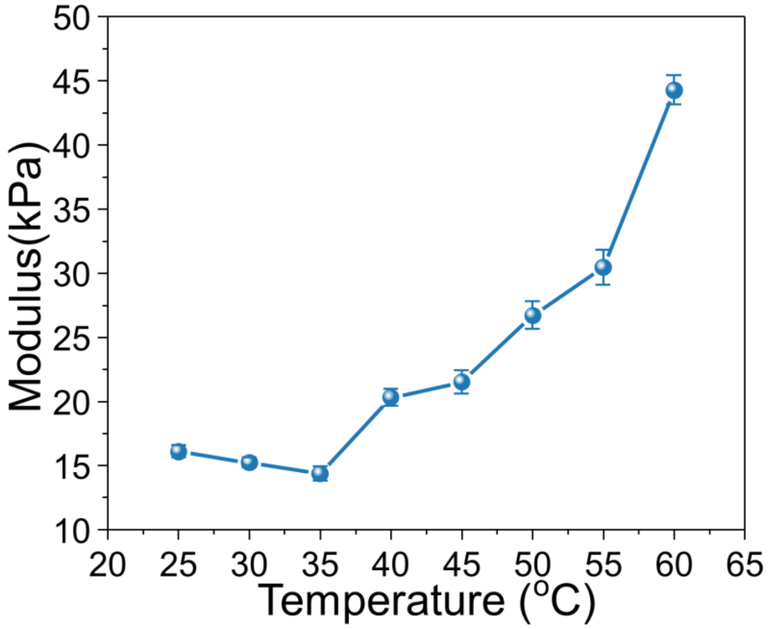

Supplementary Fig. 18 Temperature-dependent modulus of the LIHAM hydrogel. Data points are shown as mean ± s.d. (n=5).

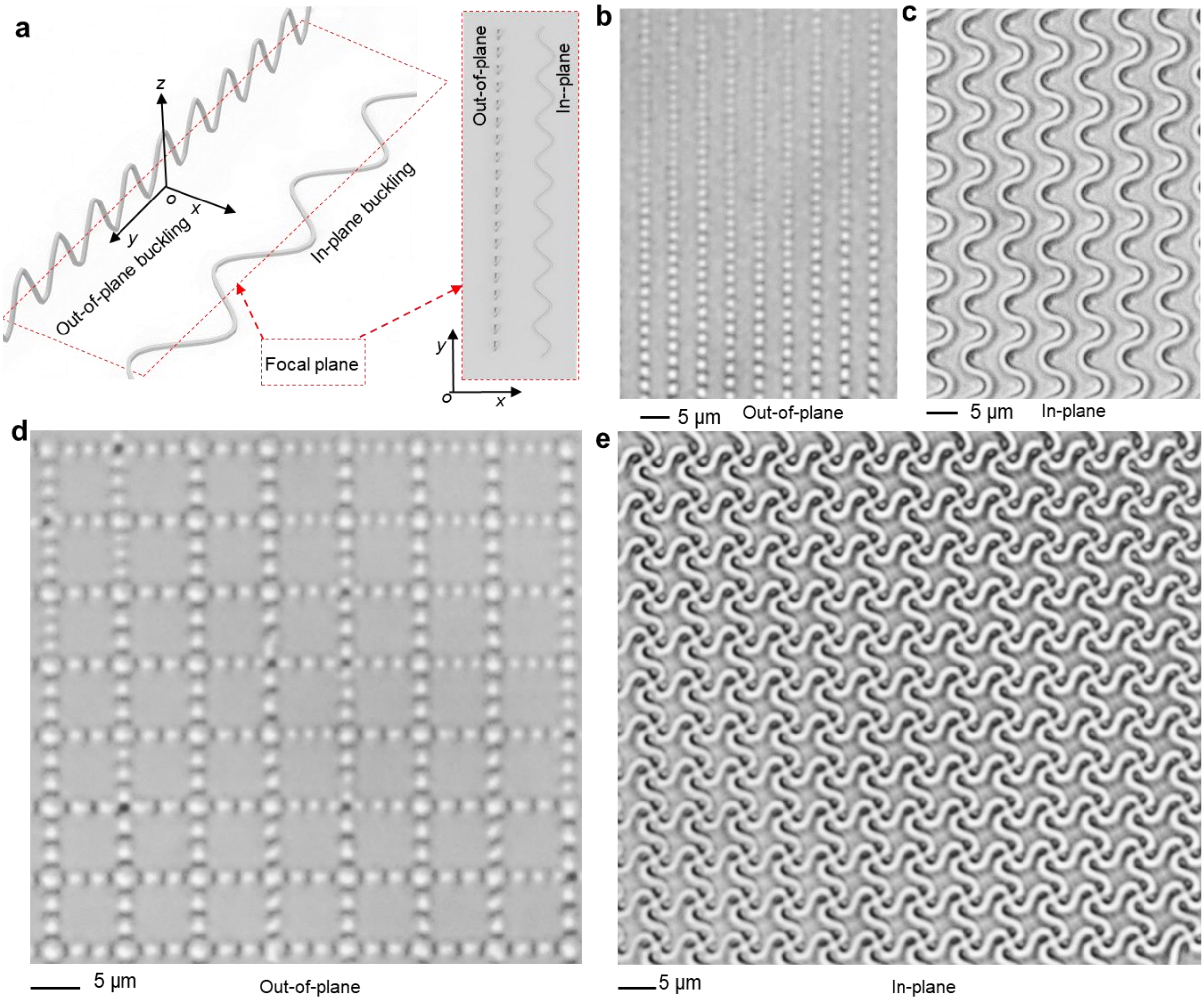

Supplementary Fig. 19 Out-of-plane and in-plane buckling modes. a, Schematic illustration of out-of-plane and in-plane buckling modes. **b**, Optical image of an actuated line array with out-of-plane buckling mode, and the arrays are printed with a low laser dosage. **c**, Optical image of an actuated line array with in-plane buckling mode, and the arrays are printed with a high laser dosage. **d**, Optical image of an actuated meta-lattice with out-of-plane buckling mode. **e**, Optical image of an actuated meta-lattice with in-plane buckling mode.

Supplementary Videos

Supplementary Video 1 Deformation of a conventional PNIPAM hydrogel during heating and cooling.

Supplementary Video 2 Deformation of a PNIPAM/PVA hydrogel during heating and cooling.

Supplementary Video 3 Deformation of a LIHAM during heating and cooling.

Supplementary Video 4 Buckling and recovery of a beam when heating and cooling.

Supplementary Video 5 Achiral transformation of a square meta-lattice (printed with 50 mW) with a hatching distance of 10 μm.

Supplementary Video 6 Chiral transformation of a square meta-lattice (printed with 20 mW) with a hatching distance of 10 μm.

Supplementary Video 7 Geometrical transformation of a triangular meta-lattice (printed with 30 mW) with a hatching distance of 10 μm.

Supplementary Video 8 3D reconstructed rotation showing geometrical transformations of a tent-like 3D architecture (IP-S).

Supplementary Video 9 Disappearing (upon heating) and recovery (upon cooling) of the *Girl with a Pearl Earring* image in metastructure (150×180 pixels^2, 27000 units) actuated by the LIHAM.
